# Observation of DNA intertwining along authentic budding yeast chromosomes

**DOI:** 10.1101/gad.305557.117

**Published:** 2017-11-01

**Authors:** Ainhoa Mariezcurrena, Frank Uhlmann

**Affiliations:** Chromosome Segregation Laboratory, The Francis Crick Institute, London NW1 1AT, United Kingdom

**Keywords:** DNA topology, chromosome segregation, sister chromatid intertwining, precatenanes, topoisomerase II, *S. cerevisiae*

## Abstract

DNA replication of circular genomes generates physically interlinked or catenated sister DNAs. These are resolved through transient DNA fracture by type II topoisomerases to permit chromosome segregation during cell division. Topoisomerase II is similarly required for linear chromosome segregation, suggesting that linear chromosomes also remain intertwined following DNA replication. Indeed, chromosome resolution defects are a frequent cause of chromosome segregation failure and consequent aneuploidies. When and where intertwines arise and persist along linear chromosomes are not known, owing to the difficulty of demonstrating intertwining of linear DNAs. Here, we used excision of chromosomal regions as circular “loop outs” to convert sister chromatid intertwines into catenated circles. This revealed intertwining at replication termination and cohesin-binding sites, where intertwines are thought to arise and persist but not to a greater extent than elsewhere in the genome. Intertwining appears to spread evenly along chromosomes but is excluded from heterochromatin. We found that intertwines arise before replication termination, suggesting that replication forks rotate during replication elongation to dissipate torsion ahead of the forks. Our approach provides previously inaccessible insight into the topology of eukaryotic chromosomes and illuminates a process critical for successful chromosome segregation.

## Abstract (precis)

Here, Mariezcurrena and Uhlmann use excision of chromosomal regions as circular “loop outs” to convert sister chromatid intertwines into catenated circles. This reveals intertwining at replication termination and cohesin-binding sites, and intertwining appears to spread evenly along chromosomes but is excluded from heterochromatin.

Genetic information is encoded in DNA as a one-dimensional sequence of bases. However, it is the three-dimensional configuration of the DNA that directs how the information is accessed. This makes DNA topology a crucial player in genome function. The topological state of cellular DNA is under the control of DNA topoisomerases—enzymes that generate transient DNA breaks, rearrange, and then religate their substrates. Topoisomerases are fundamental for the survival of all organisms and have crucial roles in virtually all DNA transactions, including DNA replication, transcription, and chromosome condensation and segregation (DiNardo et al. 1984; Holm et al. 1985; Brill et al. 1987; Uemura et al. 1987; Gartenberg and Wang 1992; Schultz et al. 1992; Spell and Holm 1994; Cuvier and Hirano 2003). They fall into two main groups: Type I topoisomerases make transient ssDNA breaks, while type II enzymes temporarily break both strands of the double helix. Both types can relax supercoiling, while type II topoisomerases provide the main activity that removes catenanes or intertwinings between intact DNA duplexes.

Early studies on the replication of the circular simian virus 40 genome in infected host cells showed that, following completion of DNA synthesis, replication products are catenated before being resolved (Sundin and Varshavsky 1980). This was rationalized by the steric exclusion of topoisomerases from DNA at the point where replisomes converge during replication termination. At these sites, topoisomerases are unable to access the last 10 or so turns of the double helix. While the exact events during replication termination are still being elucidated (Dewar et al. 2015), intertwining of the resulting replication products appears inevitable. In addition to replication termination, intertwines between sister chromatids might arise already during replication elongation. As helicases unwind the parental strands to make them accessible to polymerases, overwinding accumulates ahead of the replication fork. This positive supercoiling can be relieved by topoisomerases, although it has been proposed that some of the torsional stress is passed behind the replication fork through fork rotation and takes the shape of intertwined replication products (referred to as precatenanes) (Champoux and Been 1980). Evidence for fork rotation has been presented during prokaryotic DNA replication and during in vitro plasmid replication (Peter et al. 1998; Lucas et al. 2001; Wang et al. 2008b; Cebrián et al. 2015). Eukaryotic chromosomal replication forks are embedded into their nuclear surroundings and are thought to be organized into higher-order replication foci. Whether eukaryotic replication forks therefore rotate to relieve torsion and generate intertwines during DNA replication is not known.

While intertwines have not been directly observed along eukaryotic linear chromosomes, there is no reason to doubt that they exist and that they must be resolved during chromosome segregation. Topoisomerase II (topo II) activity is required during a successful anaphase (DiNardo et al. 1984; Uemura et al. 1987; Downes et al. 1991; Wang et al. 2008a), suggesting that DNA strand passage is needed to resolve sister chromatid intertwining during chromosome segregation. An argument has been made that differential resolution timing of different parts of the genome could even be the reason for reproducible segregation timing differences during anaphase (Vig 1981; D'Ambrosio et al. 2008a). For example, the budding yeast ribosomal DNA (rDNA) is always the last locus to segregate during anaphase, likely due to its late topological resolution. The segregation timing in turn contributes to setting up nuclear chromosome positioning in interphase (Gerlich et al. 2003) such that the budding yeast rDNA is always found in the nuclear periphery. On the other hand, chromosome resolution failure is a frequent cause for chromosome missegregation, resulting in aneuploid daughter cells. A mutation in the chromosomal condensin complex causes chromosome resolution defects and spurs neoplastic malignancies (Woodward et al. 2016), exemplifying the importance of chromosome resolution for genome stability.

Sister chromatid intertwining in eukaryotes has traditionally been studied using circular plasmids or minichromosomes in budding yeast. Based on these studies, it has become clear that catenation of these circular substrates arises as a consequence of DNA replication. Most sister circles are rapidly decatenated by topo II during and after S phase, although a certain fraction remains intertwined into mitosis. Persistence of catenanes depends on the cohesin complex that provides proteinaceous links between the sister chromatids. During mitosis, the last remaining catenanes are removed in a reaction that is promoted by condensin and by physical sister chromatid separation by the mitotic spindle (Koshland and Hartwell 1987; Baxter et al. 2011; Farcas et al. 2011; Charbin et al. 2014). The levels of the Smc5–Smc6 complex increase along chromosomes when intertwines accumulate following topo II inactivation, which led to the proposal that the Smc5–Smc6 complex recognizes sites of intertwining (Jeppsson et al. 2014). These previous studies did not address where along authentic chromosomes intertwines arise and persist or when during S phase they are generated.

Having provided a wealth of insight into DNA topology, small circular minichromosomes differ in their structure from authentic linear chromosomes. Minichromosomes are sealed topological units, which is in contrast to the open-ended nature of their much longer linear counterparts. Protein association with linear chromosomes is thought to establish topologically constrained domains, although their boundaries are likely to be dynamic. Authentic chromosomes could thus be seen as a loose topological network. The consequences of this on intertwine behavior are difficult to predict. Minichromosomes typically contain centromeres, which bestow distinct topological characteristics that might differ from chromosome arms (Díaz-Ingelmo et al. 2015). In addition, minichromosomes are up to two orders of magnitude less stable during cell division, emphasizing the importance of also studying the topology of authentic chromosomes (Koshland and Hartwell 1987).

Investigating the topology of linear eukaryotic chromosomes poses a technical challenge in that topological information is retained only in topologically closed systems. While protein factors introduce topological constraints along linear chromosomes, proteins are removed during the isolation of nucleic acids. This renders even long linear DNA molecules topologically unconstrained. In this study, we used site-specific recombination to excise regions along budding yeast chromosomes as DNA circles to make them amenable to topological analysis. This allowed us to demonstrate sister chromatid intertwining along linear chromosomes following DNA replication. Evidence for intertwining is apparent already before replication termination, suggesting that replication forks rotate during replication elongation to dissipate torsional stress. Following completion of S phase, catenanes are widely distributed along chromosomes, with the exception of heterochromatin, suggesting that they propagate relatively freely. Our approach opens new opportunities to analyze the DNA topology of eukaryotic chromosomes.

## Results

### Catenated chromosomal loop outs following DNA replication

**Figure 1.**
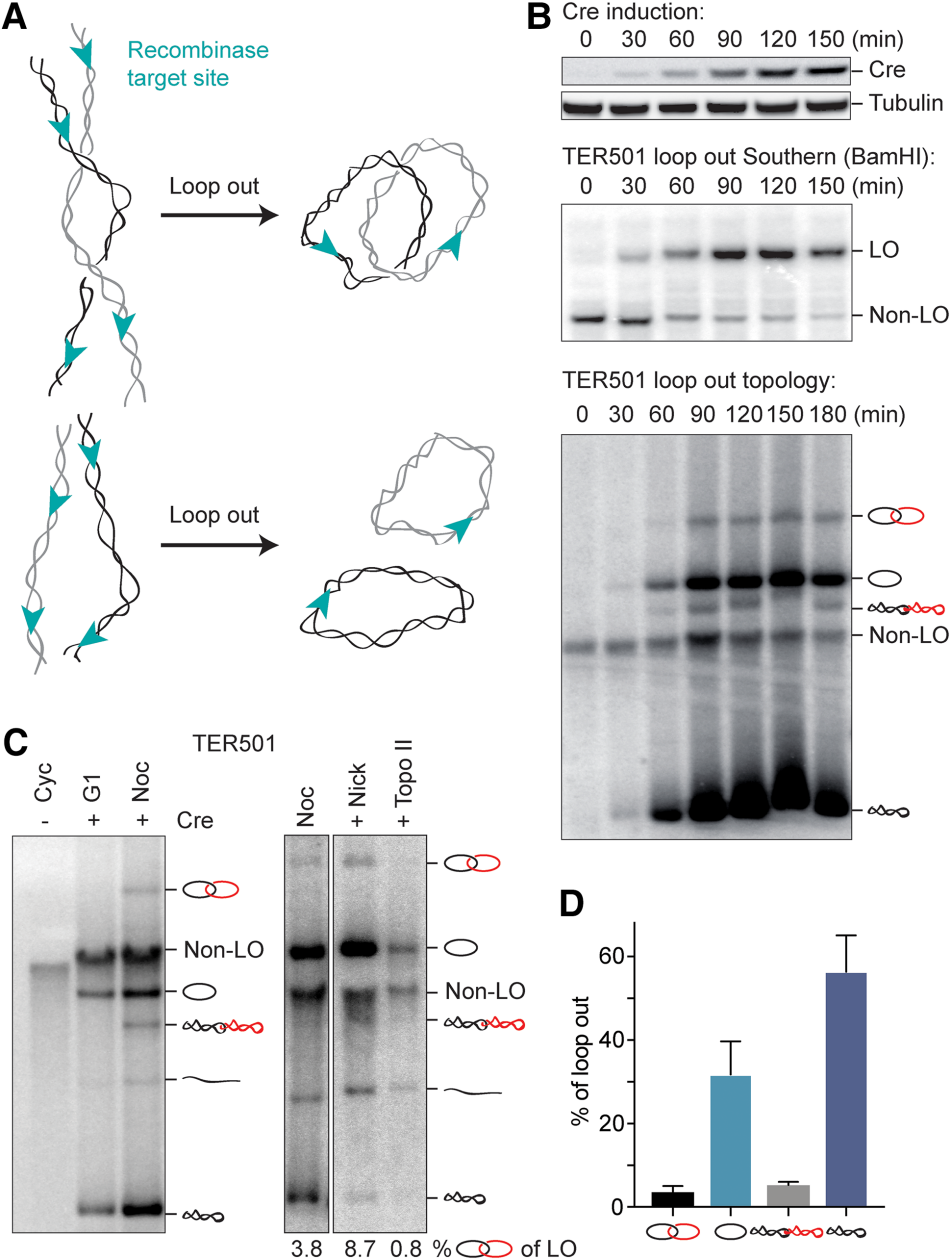
Excision of a chromosomal region in G2/M results in catenated loop outs. (*A*) Schematic to illustrate the possible outcomes of excising a chromosomal region as circular loop outs. Intertwines between sister chromatids might result in catenated loop outs. (*B*) Time course of Cre recombinase induction in G2/M-arrested cells. The Western blot shows Cre levels; tubulin served as a loading control. A Southern blot following BamHI digestion confirms Cre-mediated excision of the TER501 region flanked by the two *loxP* sites (cf. Supplemental Fig. S1A for a genomic map). Electrophoresis of the untreated DNA samples reveals a number of topoisomers. (*C*) Topological analysis of loop outs (LOs) from G1-arrested and G2/M (Noc)-arrested cells together with enzyme treatments to assign band identities. The fraction of nicked catenanes as a percentage of all loop out species is indicated. Note that non-loop out DNA (non-LO) migrates at the exclusion limit for linear DNAs, and the relative position of circular species changes depending on the length of the gel run. (Nick) Nicking enzyme Nt.Bpu10I. (*D*) Quantification of the indicated topoisomers as a percentage of all loop out species from the four last time points following Cre induction in *B*. The means ± standard error are shown.

To study local chromosome topology along authentic budding yeast chromosomes, we used site-specific recombination between tandem recombinase recognition sites to excise covalently closed circles or “loop outs” (Fig. 1A; Supplemental Fig. S1A). We introduced *loxP* sites flanking a 17-kb region on the left arm of chromosome V, surrounding the replication termination site TER501, where replication forks from the two efficient replication origins ARS507 and ARS508 converge (McGuffee et al. 2013). Cells were arrested in G2/M by nocodazole treatment, and then we induced Cre recombinase expression from the galactose-inducible *GAL1* promoter. Loop out was confirmed by Southern blotting and occurred in about two-thirds of cells within 90 min after induction, after which it reached a plateau (Fig. 1B). In subsequent experiments, unless otherwise stated, we collected samples for DNA extraction 90 min after recombinase induction.

We next analyzed the topology of the loop outs by resolving purified DNA by native agarose gel electrophoresis. A band corresponding to the chromosomal DNA before loop out was visible throughout the time course. Following loop out, a pattern of additional bands appeared, reminiscent of the topoisomers seen previously during the analysis of circular minichromosomes (Fig. 1B; Charbin et al. 2014). To assign band identities, we used two enzyme treatments. Incubation with the nicking endonuclease Nt.Bpu10I resolved the isoforms into two bands with increased intensity, corresponding to open circular forms. The slower migrating of these two bands was identified as nicked catenated species following incubation with purified human topo IIα, which resolved the catenanes to generate monomers (Fig. 1C). The fraction of catenated species was ∼10% of the total loop outs (Fig. 1D). A similar pattern of topoisomers was seen following excision of another replication termination region, TER301 (Supplemental Fig. S1B).

To address whether catenated loop outs are a reflection of sister chromatid intertwining in G2/M-arrested cells, we performed a number of experiments, described below. First, if catenated loop outs arise from intertwined sister chromatids, they should not be seen before DNA replication. As shown in Figure 1C, loop out occurred equally efficiently in G1-arrested cells, but only monomer forms became detectable at this cell cycle stage. This suggests that catenated loop outs arise as a consequence of DNA replication.

Cre recombinase acts on pairs of identical target sites, and these are preserved following recombination. It therefore could be that recombination between independently looped-out sister circles leads to their fusion or catenation. To exclude this possibility, we used the unidirectional ΦC31 integrase (Thorpe et al. 2000). ΦC31 mediates recombination between two distinct recognition sites: *attB* and *attP*. Following recombination, these sites are converted into *attL* and *attR* sites that are no longer a substrate for ΦC31. Loop out between tandem *attB* and *attP* sites flanking the replication termination region TER603 on chromosome VI following ΦC31 expression occurred with similar efficiency and generated catenated and monomeric circles in a comparable ratio compared with Cre-mediated loop out of the same region (Supplemental Fig. S1C,D). This suggests that catenated loop outs are not the consequence of sequential recombination reactions between initially unconnected circles.

### Catenated loop outs record physiological changes of intertwining

**Figure 2.**
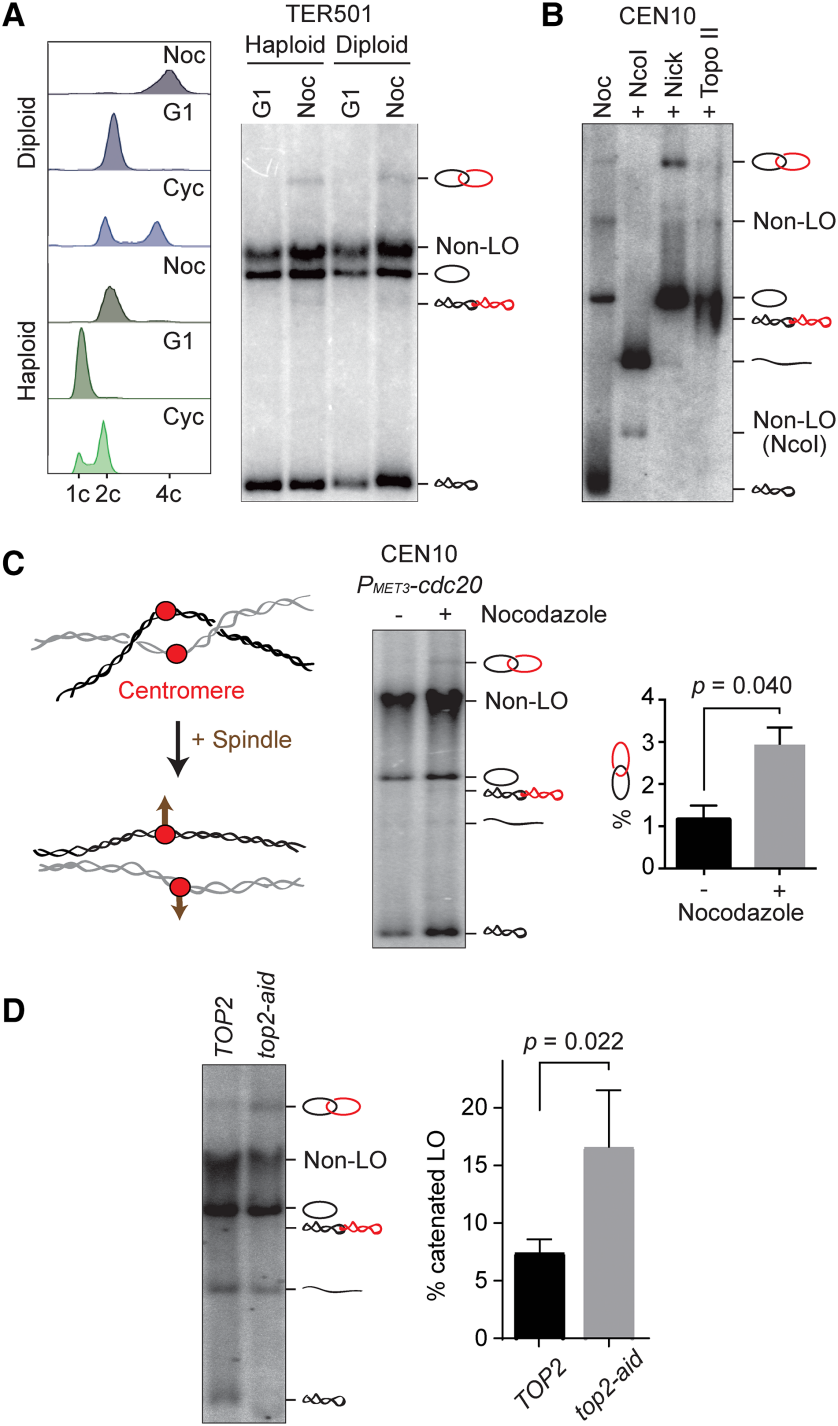
Catenated loop outs reflect the expected chromosome topology. (*A*) Comparison of loop out topologies in haploid and diploid cells following excision during α-factor arrest in G1 or a nocodazole-induced (Noc) arrest in G2/M. FACS analysis of DNA content to confirm ploidy and cell cycle stage as well as the gel blot are shown. Exponentially cycling cells (Cyc) served as a comparison. (*B*) Excision of a region surrounding the centromere of chromosome X (CEN10) generates catenated loop outs. Electrophoresis of the DNA sample following excision is shown next to the indicated enzyme treatments. (*C*) Schematic of how the mitotic spindle is expected to affect centromere topology as well as a gel image of the loop outs generated during G2/M arrest due to Cdc20 depletion in the presence or absence of nocodazole. The quantification shows the relative abundance of the open catenated loop out in three independent repeats of the experiment. The means ± standard error are shown. An unpaired *t*-test confirmed a statistically significant increase of catenated loop outs in the presence of nocodazole. (*D*) Augmented catenated loop outs following topo II down-regulation. A gel image is shown of loop outs after cells progressed through S phase with wild-type (*TOP2*) or reduced (*top2-aid*) levels of topo II. The quantified means ± standard error from three independent experiments are shown. An unpaired *t*-test confirmed a statistically significant increase of catenated loop outs following topo II depletion.

An important question remains whether catenated loop outs reflect the intertwining of sister chromatids before loop out or whether catenation might have arisen after loop out. To address this, we compared loop out catenation in a haploid and a diploid strain arrested in G1. We used a *MAT***a**/*MAT***a** diploid strain that can be arrested in G1 using α-factor treatment and in which TER501 was flanked by *loxP* sites on both copies of chromosome V. If excised DNA circles had a propensity to catenate following loop out, we would expect to detect catenanes following excision of two circles in the diploid strain in G1. However, this was not the case (Fig. 2A). As in the haploid strain, catenated circles were observed in the diploid strain only following DNA replication in G2/M. This is consistent with the possibility that catenated loop outs reflect the intertwining of sister chromatids.

As an additional test of whether catenated loop outs reflect sister chromatid intertwining, we inserted tandem *loxP* sites flanking a 12.3-kb region encompassing the centromere of chromosome X (CEN10) (Supplemental Fig. S2A). As before, excision in cells arrested in G2/M by nocodazole treatment revealed catenated loop outs at this locus (Fig. 2B). Under tension of the mitotic spindle, sister centromeres undergo dynamic separation and rejoining (“centromere breathing”) (Goshima and Yanagida 2000). As a consequence of their physical separation, intertwining between sister centromeres should be reduced. To test whether this is the case, we arrested cells in G2/M by depletion of the anaphase-promoting complex activator Cdc20, which allows chromosomes to biorient on the mitotic spindle and centromere breathing to commence. For comparison, we used an aliquot of the same culture arrested by Cdc20 depletion but also added nocodazole to depolymerize the mitotic spindle. This comparison revealed that the presence of a mitotic spindle significantly reduced the levels of catenated loop outs (Fig. 2C), consistent with them reflecting the levels of sister chromatid intertwining.

Next, we aimed to increase intertwine retention following DNA replication by depleting topo II. We reduced topo II levels by replacing its endogenous promoter with a methionine-repressible *MET3* promoter and fusing it to an auxin-inducible degron. We synchronized cells in G1 by α-factor treatment and repressed topo II expression before releasing cells to progress through S phase and into a nocodazole-imposed G2/M arrest. Topo II was virtually undetectable by Western blotting, yet its functional depletion was incomplete under these conditions, as cells successfully concluded chromosome segregation in a parallel culture without nocodazole and ceased proliferation only after two to three cell divisions (Supplemental Fig. S2B). Nevertheless, reduced topo II levels resulted in a markedly increased fraction of catenated loop outs at TER501 (Fig. 2D). Thus, an expected increase of sister chromatid intertwines manifests itself in an increased fraction of catenated loop outs, suggesting that the former gave rise to the latter.

Topo II can both decatenate as well as catenate DNA circles (Vologodskii et al. 2001). We therefore performed a final test of whether catenated loop outs might arise after excision of unconnected DNA circles by the catenating action of topo II. We again arrested cells in G2/M by nocodazole treatment but now inactivated topo II using the temperature-sensitive *top2-4* allele by shifting the culture to 37°C before loop out. *top2-4* was rapidly inactivated following temperature shift (Supplemental Fig. S2B; Holm et al. 1985), although we did not use this allele in the previous experiment because persistence of inactive Top2-4 protein on chromosomes could interfere with DNA replication (Baxter and Diffley 2008). Topo II inactivation in G2/M cells before Cre recombinase induction did not reduce the fraction of catenated loop outs (Supplemental Fig. S2C). This suggests that catenated loop outs result from intertwining that existed between sister chromatids before excision. We note that the fraction of catenated loop outs also did not increase following topo II inactivation. By the same argument, catenation between loop outs therefore was not lost in a major way through topo II action following excision. Therefore, the fraction of catenated loop outs reflects the frequency of intertwining between sister chromatids before excision.

### Intertwines are found at replication termination regions but also at replication origins

**Figure 3.**
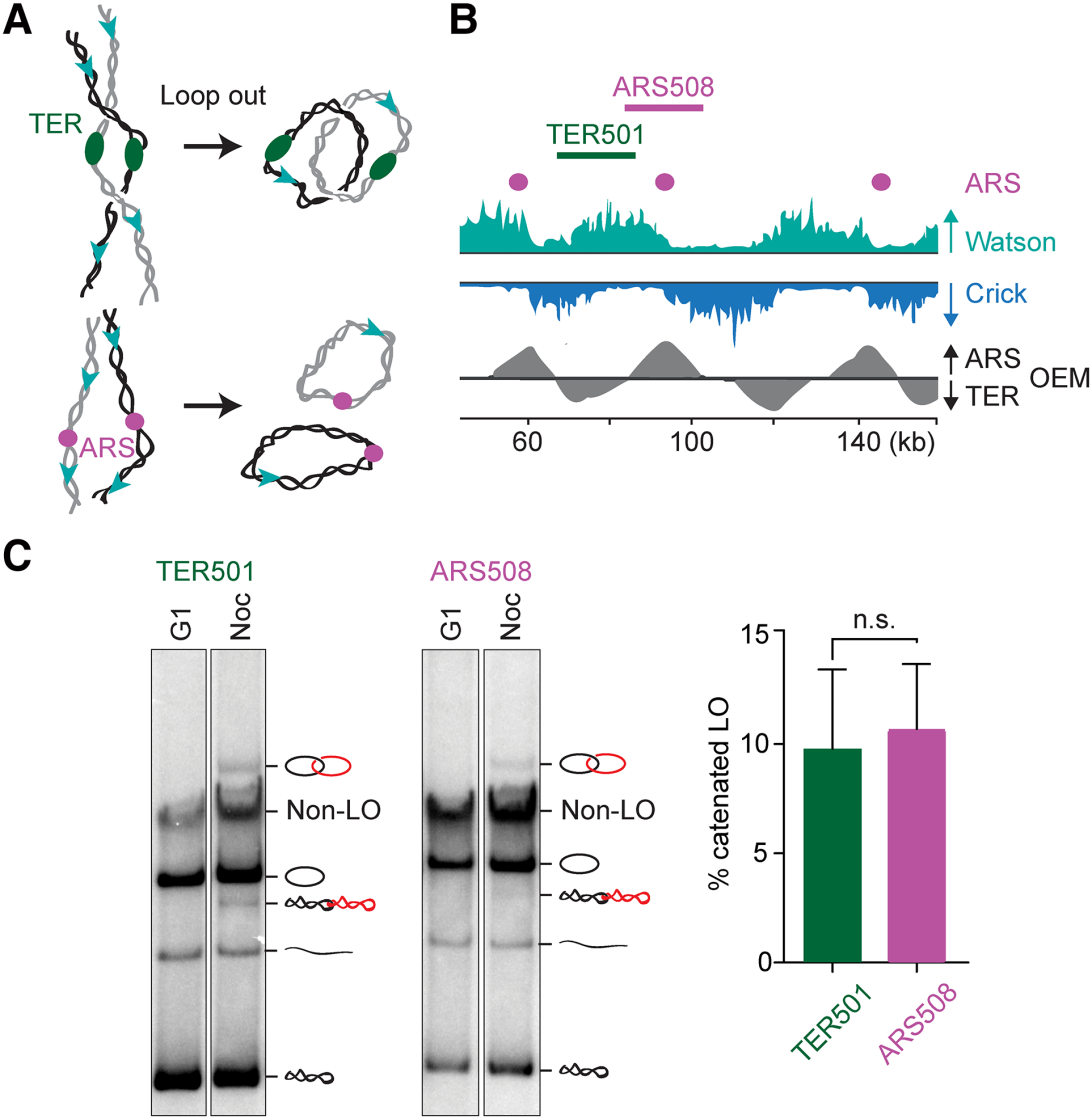
Intertwines at replication termination regions as well as origins. (*A*) Schematic of sister chromatid intertwines at replication termination (TER) regions and origins (ARS). (*B*) Okazaki fragment directionality (McGuffee et al. 2013) along the investigated region of chromosome V. The origin efficiency matrix (OEM) indicates the positions of replication origins and regions of replication termination. (*C*) Topology comparison of the TER501 and ARS508 loop outs as well as quantification of the fraction of catenated loop outs from four independent repeats of the experiment. The means ± standard error are shown. An unpaired *t*-test found no significant difference (n.s.) between the levels of catenated loop outs at TER501 and ARS508.

So far, for topological analyses, we mostly excised replication termination regions, where intertwines are thought to arise. We next wanted to analyze a region where intertwines are less likely to be generated. We chose an efficient replication origin, where topological torsion due to DNA replication is expected to be directed away in both directions (Fig. 3A). Based on Okazaki fragment directionality (McGuffee et al. 2013), we selected ARS508 as a strong replication origin adjacent to TER501 (Fig. 3B). Following Cre recombinase-mediated excision of similarly sized regions surrounding TER501 or ARS508, we were surprised to find that the proportion of catenated loop outs was equal at both places (Fig. 3C). Thus, catenanes are not restricted to regions of replication termination. This result could be explained if intertwines form all along chromosomes during DNA replication. Alternatively, catenanes might preferentially arise during replication termination but are able to redistribute.

### Cohesin retains intertwines but does not restrict their location

**Figure 4.**
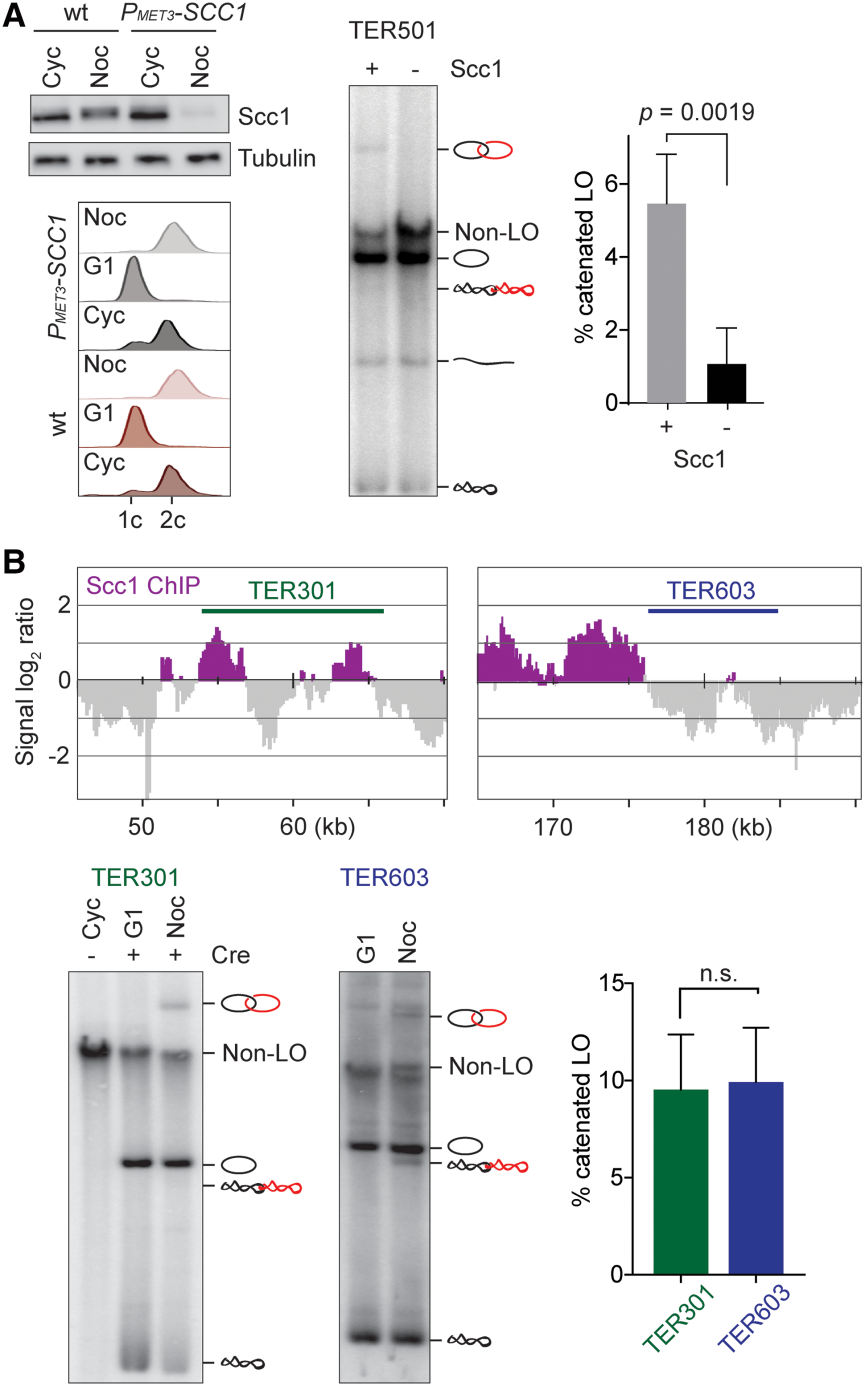
Cohesin maintains intertwines but does not restrict their location. (*A*) A region surrounding TER501 was excised in G2/M-arrested cells in the presence of cohesin or after the cohesin subunit Scc1 was depleted by *MET3* promoter repression following release from synchronization in G1. FACS analysis of DNA content confirmed cell synchrony, and a Western blot shows Scc1 depletion together with a gel image to analyze loop out topology. The quantification shows the means ± standard error of the fraction of catenated loop outs from four independent repeats of the experiment. An unpaired *t*-test revealed a significant reduction in catenated loop outs in the absence of cohesin. (*B*) Topoisomer distribution of excised replication termination regions TER301 and TER603 enriched for or devoid of cohesin, respectively. The cohesin distribution is depicted (Ocampo-Hafalla et al. 2007), and the fraction of catenated loop outs was quantified in four independent repeats of the experiment. The means ± standard deviations are shown. An unpaired *t*-test found no significant difference in the fraction of catenated loop outs from the two loci.

Based on studies with circular minichromosomes, it is thought that the chromosomal cohesin complex is required to retain intertwines between replicated sister chromatids (Farcas et al. 2011; Charbin et al. 2014). We note that both the TER501 and ARS508 regions, analyzed above, contain cohesin-binding sites (Supplemental Fig. S3A). We therefore addressed the role of cohesin in maintaining intertwines along authentic chromosomes. We used cells in which expression of the cohesin subunit Scc1 could be repressed under control of the *MET3* promoter. Owing to Scc1's inherent instability during anaphase and G1, it is efficiently depleted following transcriptional repression (Fig. 4A). After synchronization in G1 using α factor and a shift to methionine-containing medium, cells were released and arrested in G2/M by nocodazole treatment. Cre recombinase-mediated excision of TER501 revealed a markedly reduced fraction of catenated loop outs in cohesin-depleted cells compared with control cells (Fig. 4A). This suggests that, as in the case of circular minichromosomes, the physical proximity conferred by sister chromatid cohesion supports the retention of sister chromatid intertwining along linear chromosomes.

If cohesin is required to retain sister chromatid intertwining, do intertwines accumulate at cohesin-binding sites? The Smc5/Smc6 complex, implicated in marking intertwines, is also found at cohesin-binding sites (Jeppsson et al. 2014). To address this, we excised two comparable replication termination regions on chromosomes III (TER301) and VI (TER603). The TER301 region contains two cohesin-binding sites, while sequences surrounding TER603 are largely free of cohesin (Fig. 4B; Ocampo-Hafalla et al. 2007). Following excision in G2/M-arrested cells, the fraction of catenated loop outs was comparable at both locations. Thus, cohesin promotes retention of intertwines by maintaining proximity between sister chromatids but does not restrict intertwines to cohesin-binding sites.

While cohesin promotes intertwine retention, condensin has been implicated in their removal (Baxter et al. 2011; Charbin et al. 2014). We therefore excised two replication termination regions—TER404 and TER702—that are relatively enriched for or depleted of condensin (Supplemental Fig. S3B; D'Ambrosio et al. 2008b). Again, we could not detect a significant difference in the fraction of catenated loop outs at these two locations. The fraction of catenated loop outs in G2/M-arrested cells also remained unchanged following condensin depletion (Supplemental Fig. S3C). This suggests that condensin does not affect the level of intertwining along chromosomes at the time when cells enter mitosis, consistent with what has been observed using circular minichromosomes (Farcas et al. 2011; Charbin et al. 2014). It will be important to investigate whether condensin contributes to intertwine resolution during anaphase, especially at the budding yeast rDNA locus, whose segregation depends mostly on condensin (D'Ambrosio et al. 2008a). This could clarify the nature of anaphase bridges that are so characteristic for condensin-compromised cells.

### Intertwines are excluded from heterochromatin

**Figure 5.**
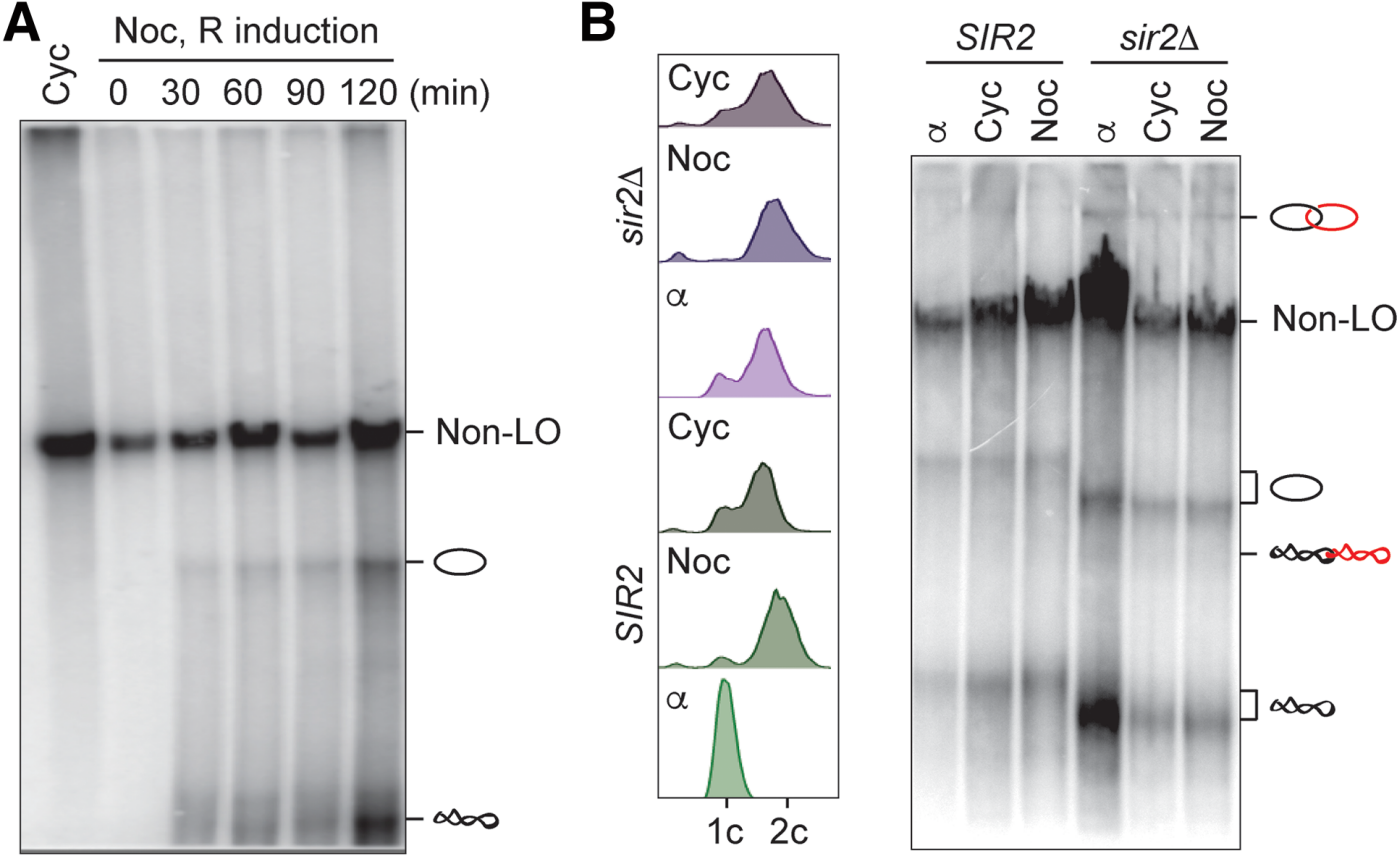
Heterochromatin excludes intertwines. (*A*) Loop out topology was analyzed over a time course of *HMR* locus excision following R recombinase induction in G2/M (Noc)-arrested cells. A sample of exponentially cycling (Cyc) cells before recombinase induction is included for comparison. (*B*) Comparison of topoisomer distributions of the *HMR* loop out in wild-type and *sir2*Δ cells treated as indicated with α factor (α) or nocodazole (Noc). Cell cycle stages were confirmed by FACS analysis of DNA content. We note that loop outs from the *sir2*Δ strain migrate distinctly faster, which could be caused by a different supercoiling status, although we do not know the reason.

With the objective to compare the topological behavior of different chromosomal regions, we analyzed intertwining along the transcriptionally silent *HMR* mating type locus. This locus is covered by the Sir2–Sir4 silencing complex, including the Sir2 histone deacetylase that maintains histones in a hypoacetylated state. To excise this region, we used a strain in which the locus is flanked with target sites for the R recombinase (Chang et al. 2005). Both R and Cre recombinases belong to the same group of bidirectional tyrosine recombinases. Induction of R recombinase expression in G2/M-arrested cells led to excision of the *HMR* locus (Fig. 5A). However, we saw only monomeric species of the loop out and could not detect any catenated forms.

Given the absence of catenated loop outs, we wanted to ascertain whether we would be able to detect catenanes if there were any. To do this, we excised the *HMR* locus during an α-factor-induced G1 arrest and then released cells to pass through a synchronous cell cycle before rearrest in the following G1 (Supplemental Fig. S4). At late time points during S phase, when the excised *HMR* circles are expected to replicate (Hawkins et al. 2013), catenated species became detectable, and these were resolved during mitosis before cells returned to G1. This suggests that intertwines form during replication of the *HMR* locus that become visible if the locus is excised as a topologically closed circle before DNA replication. In contrast, intertwines are not retained along the *HMR* locus following its replication as part of the authentic chromosome III.

An explanation for intertwine exclusion from the *HMR* locus might lie in its distinct chromatin state. The Sir2–Sir4 complex covers this region and might introduce a chromatin property that disfavors intertwining. To examine whether its heterochromatic state caused intertwine exclusion, we repeated *HMR* locus excision in a *sir2*Δ strain in which silencing is abolished. As a consequence of silent mating type locus derepression, *sir2*Δ cells no longer respond to α-factor treatment (Fig. 5B). Catenanes became clearly discernible in *sir2*Δ cells growing asynchronously or treated with α-factor or nocodazole. Therefore, the transcriptionally repressive chromatin state at *HMR*, possibly a more rigid chromatin structure due to Sir2–Sir4 complex binding, disfavors the persistence of intertwines in this region.

### Intertwines arise during DNA replication

**Figure 6.**
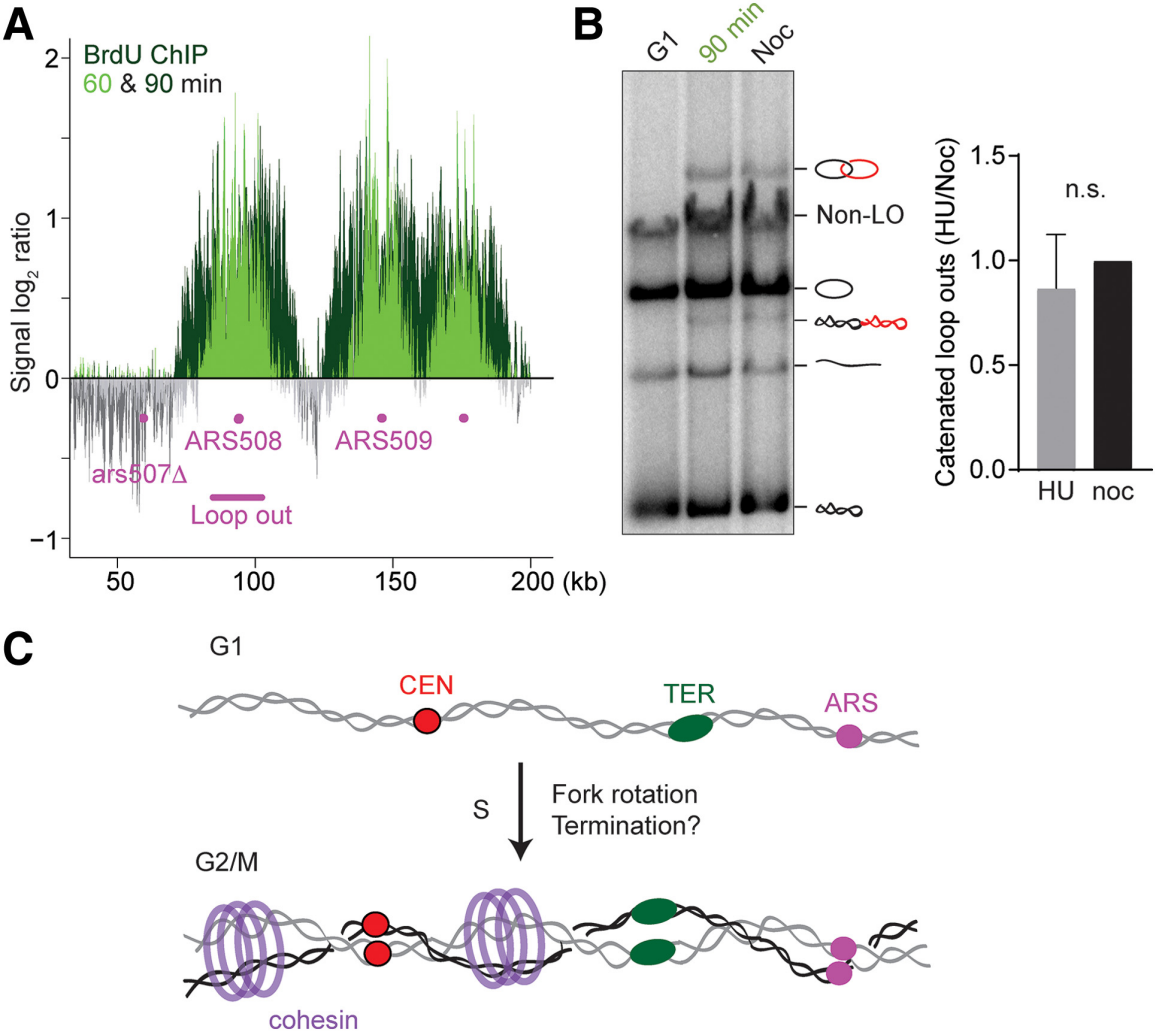
Intertwines arise during DNA replication elongation. Cells were released from an α-factor-induced G1 block into medium containing 0.1 M HU, and galactose was added 30 min later to induce Cre recombinase expression. (*A*) BrdU incorporation at 60 and 90 min was analyzed by BrdU immunoprecipitation as a marker for replication fork progression. (*B*) The loop out topology of a region containing ARS508 was analyzed in samples taken from the G1 arrest, 90 min in HU, and cells released from G1 to progress into a nocodazole-imposed G2/M arrest (Noc). The fraction of catenated loop outs was quantified in four independent repeats of the experiment and were normalized to the fraction in G2/M. The means ± standard error are shown. An unpaired *t*-test revealed no significant difference between the two conditions. (*C*) A model for catenane formation and distribution along eukaryotic chromosomes. Proximity between sister chromatids, maintained by the cohesin complex, allows retention of sister chromatid intertwining but does not restrict intertwine distribution along chromosomes.

Next, we addressed whether intertwines arise during replication elongation or are the consequence of replication termination. We synchronized cells in G1 using α-factor treatment and released them to progress through S phase in the presence of low concentrations of hydroxyurea (HU; 0.1 M) to slow down replication fork progression. DNA replication during this experiment was visualized by incorporation of bromodeoxyuridine (BrdU) into newly synthesized DNA followed by DNA immunoprecipitation using an α-BrdU antibody (Fig. 6A). Thirty minutes following α-factor release, galactose was added to induce Cre recombinase expression to excise a region surrounding ARS508. To minimize nearby termination events, we used a strain in which we had deleted the neighboring ARS507. At 60 min, replication had passed through the region marked for excision, and the first loop out products started to become detectable (Supplemental Fig. S5A). After 90 min, replication had slowly progressed, but, based on the BrdU incorporation pattern, replication forks had not converged, and replication termination events had not yet occurred. We then took a final sample to analyze loop out topology. Catenated loop outs were clearly discernible at this time (Fig. 6B), suggesting that intertwining of sister chromatids arises during replication elongation and prior to termination, at least in cells undergoing S phase in the presence of HU.

When we quantified the fraction of catenated loop outs at 90 min in HU, we found that their abundance was similar to what is seen following excision in G2/M (Fig. 6B). This was surprising, as we would expect additional intertwines to arise during the remainder of DNA replication and during replication termination. The apparently unchanged levels of intertwines could be explained if they reflected a constant steady-state level of intertwine retention rather than the numbers of their initial production. Similar to what is seen on circular minichromosomes, most intertwines generated along linear chromosomes might be readily resolved by topo II, while a lower steady state of intertwines persists.

To investigate the relationship between intertwine generation and persistence, we created a situation in which intertwine formation is directed to a specific region close to ARS508. Replication termination events are rare in the vicinity of this active origin (McGuffee et al. 2013). We changed this by insertion of a replication fork barrier (RFB), which usually enforces unidirectional replication fork arrest in the rDNA (Supplemental Fig. S5B; Kobayashi 2003). The arrested fork then awaits an oncoming fork from ARS509, with replication termination occurring in a majority of cells at this RFB. Despite the expected localized intertwine generation during termination at the RFB, the fraction of catenated loop outs in G2/M-arrested cells again remained unchanged (Supplemental Fig. 5C). This suggests that local intertwine generation is offset by their resolution and dispersion along chromosomes, resulting in a low and largely uniform level of intertwine retention.

## Discussion

Despite its importance for almost all aspects of chromosome function, DNA topology of authentic eukaryotic chromosomes remains an understudied field. Here, we developed an approach to analyze sister chromatid intertwining between replicated yeast chromosomes. Excision of chromosomal regions as circles retains local topological information and allows its visualization. Using this method, we found that intertwines arise in S phase already during replication elongation, consistent with the notion that torsion ahead of the replication fork is in part passed on by fork rotation. Fork rotation emerges as a plausible mechanism that could likewise facilitate DNA unwinding and replication of the final turns of the double helix during replication termination (Dewar et al. 2015). Fork rotation during replication elongation is expected to generate intertwines with little sequence specificity over wide parts of the genome. Consistently, we found that intertwines that are retained on chromosomes when cells enter mitosis are widely distributed with little preference for specific locations. While intertwine retention depended on sister chromatid proximity provided by the cohesin complex, the location of the intertwines was not restricted by cohesin (Fig. 6C).

The frequency of intertwining that we detected in G2/M-arrested cells was around one-tenth of excised loop outs in cells grown in rich medium. The frequency was consistently somewhat lower in all experiments that used cells grown in synthetic medium lacking methionine to maintain gene expression from the *MET3* promoter, although we do not know the reason for this difference. Loop outs spanned 10–17 kb in length. If catenated loop outs are a true reflection of intertwining along yeast chromosomes, this corresponds to one intertwine for roughly every 150 kb. Considering a median budding yeast replicon size of ∼50 kb (Hawkins et al. 2013), we would expect three replication termination events to have taken place in a chromosome section of this size. Even the lowest estimate of how many intertwines are generated during termination, with additional intertwines accruing during replication elongation, makes it clear that only a small fraction of initially generated intertwines persists following DNA replication. The majority of intertwines appears to be readily resolved by topo II. Given the linear nature of yeast chromosomes, a fraction of intertwines might simply swivel off the end of chromosomes, similar to how supercoiling dissipates off chromosome ends (Joshi et al. 2010). If DNA relaxation ahead of the fork is impeded (e.g., by topo I depletion), more intertwines might be transferred behind the fork. Whether this changes the steady-state frequency of sister chromatid intertwining will be interesting to explore.

If most intertwines are readily resolved, what singles out the ones that persist? We can imagine two scenarios. Persisting linkages might possess a topology that is more complex than simple intertwines. They might, for example, be knots that have been observed as a consequence of DNA replication (Sogo et al. 1999). These might require specific activities—e.g., of the mitotic condensin complex—to resolve. Alternatively, persisting intertwines might reflect a topological steady state of intermingling between sister chromatids that are held in proximity by the chromosomal cohesin complex. This latter possibility is supported by the observation that the majority of persisting intertwines resolves upon cohesin removal.

In the search for locations where intertwines persist, we came to realize that, with very few exceptions, intertwines appear evenly distributed along chromosomes. This might come unexpected at first. However, if we consider how rapidly chromatin moves within the nucleus (Heun et al. 2001), intertwines will be hard to restrict to specific chromosomal locations. Plectonemic structures show fast motility along DNA in vitro (van Loenhout et al. 2012), and we imagine that intertwines are equally motile along chromosomes in vivo. An additional argument for the ability of intertwines to dissipate along chromosomes is the observation that small chromosomes in budding yeast are able to separate even without topo II (Spell and Holm 1994), which should be possible only if intertwines are able to slide off chromosome ends.

We found two locations from which intertwinings were excluded. First, intertwines disappeared from a centromere under tension of the mitotic spindle. This suggests that physical separation is a driving force for intertwine resolution. The second instance is the silent mating type locus, where intertwine exclusion depended on the heterochromatic nature of the locus. The Sir2–Sir4 complex associates with this region and might alter its flexibility (Bi and Broach 1997). If intertwines are diffusible along chromatin, then stiffening of a section should indeed result in the observed intertwine exclusion. Heterochromatin assembly in other eukaryotes involves heterochromatin protein (HP1) recruitment to methylated histone tails, which might equally stiffen chromatin by protein complex assembly. Notably, intertwines are thought to persist within mammalian centromeric heterochromatin, which manifests as ultrafine bridges during anaphase (Wang et al. 2008a). While the short budding yeast mating type locus excludes intertwines, this might be impossible along the vastly expanded heterochromatin surrounding mammalian centromeres. On the contrary, in this case, heterochromatin might hinder the spread of intertwines, thus effectively trapping them. Heterochromatin topology and its implications for chromosome segregation in organisms other than budding yeast will be important to investigate.

Is there a role for intertwines between sister chromatids after DNA replication? Given that most of these intertwines readily resolve following removal of the cohesin complex, there appears to be little that protects their removal other than the proximity of sister chromatids. However, the fact that these intertwines are easily removed does not exclude a possible function while they persist. Topo II has the ability to simplify topology below the thermodynamic equilibrium (Vologodskii et al. 2001). Intertwining along authentic budding yeast chromosomes might well rest below equilibrium levels, consistent with the very low numbers of intertwines that persist. On the other hand, in both bacteria and along the budding yeast rDNA, persistent intertwining impacts on segregation timing (D'Ambrosio et al. 2008a; Wang et al. 2008b). This suggests that, at least in specific cases, levels of intertwining are regulated to achieve biological purposes. Recently, evidence has been provided for a mechanism by which the transcription machinery controls topo I activity to adjust DNA topology throughout the transcription cycle (Baranello et al. 2016). The regulation of topo II, a protein targeted by numerous post-translational modification within its regulatory C-terminal domain, merits further exploration. Our assay system to investigate intertwining along authentic eukaryotic chromosomes provides a tool that should facilitate these efforts.

## Materials and methods

### Yeast strains and culture

The *Saccharomyces cerevisiae* strains used in this work were of the w303 background and are listed in Supplemental Table S1. Cre recombinase target sites were inserted into the genome by gene targeting using PCR products. A *URA3* gene flanked by short repeat sequences was used as a selectable marker (Reid et al. 2002), which was lost by counterselection on 5-fluoroorotic acid-containing medium following successful recombinase target site insertion (Rose et al. 1990). Regions flanked by recombinase target sites were selected to contain at least one essential gene to disfavor spontaneous loop out during cell expansion. All target site insertions were confirmed by DNA sequencing. Details of their genomic locations are in Supplemental Table S2. A centromeric plasmid with the Cre recombinase gene under the control of the galactose-inducible *GAL1* promoter (obtained from the Yeast Resource Center) was then introduced. Cells were maintained on complete synthetic medium lacking leucine to select for recombinase plasmid retention and then grown for experiments in rich YP medium supplemented with 2% raffinose or in complete synthetic medium lacking methionine to control Cdc20, Scc1, topo II, or Brn1 expression. Recombinase expression under the control of the *GAL1* promoter was induced by 2% galactose addition. Cells were arrested in G1 by adding 0.4 µg/mL pheromone α factor for 2–2.5 h (O'Reilly et al. 2012). Cells were released into fresh medium following filtration. To slow down DNA replication, 0.1 M HU was included in the medium. Arrest in metaphase was achieved by addition of 5 µg/mL nocodazole or depletion of Cdc20 under the control of the methionine-repressible *MET3* promoter. For Scc1 depletion, its endogenous gene promoter was replaced with the *MET3* promoter, and cells were released following synchronization in G1 into YP medium supplemented with 2 mM methionine. To deplete topo II, the *TOP2* gene was fused to an auxin-inducible degron tag (Nishimura et al. 2009). In addition, its endogenous gene promoter was replaced with the *MET3* promoter. Cells were grown in medium lacking methionine and shifted to methionine-containing medium, including 1 µM auxin indole-3-acetic acid (IAA), 2 h before release from α-factor block. To create a *MAT***a**/**a** diploid strain in which both copies of TER501 were flanked by *loxP* sites, we used HO expression to induce mating type switching and consequent diploidization of the haploid parental strain. The *MAT*α locus of the resultant MAT**a**/α diploid was then replaced with *MAT***a** by gene targeting, as described (van Werven et al. 2012). Recognition sites for the ΦC31 integrase (Thorpe et al. 2000) were inserted into the budding yeast genome by gene targeting using PCR products. For ΦC31 integrase expression, a synthetic yeast codon-optimized gene encoding the integrase (GeneArt, ThermoFisher) fused to a Pk epitope tag was cloned under the control of the galactose-inducible *GAL1* promoter into the yeast shuttle vector YCplac111, analogous to the vector used for Cre recombinase expression.

### Genomic DNA purification and analysis

Genomic DNA purification was performed following a previously described protocol (Charbin et al. 2014). DNA samples were resolved on 0.4%–0.5% agarose/TAE gels at 2 V/cm for 18–24 h. Southern transfer and hybridization were performed as described (Charbin et al. 2014). Blots were exposed to PhosphorImager screens, which were scanned using a Typhoon 9400 Imager (GE Healthcare). Quantification of band intensities was performed using ImageJ. Enzyme treatments to verify the nature of the observed bands were performed using the indicated enzymes and reaction buffers, provided by their suppliers. Restriction endonucleases were from New England Biolabs, and digests were incubated for at least 2 h at 37°C. Nicking enzyme Nt.Bpu10I (ThermoFisher) treatment was for 30 min, while human topo IIα (Topogen) treatment was for 1 h, both at 37°C.

### Yeast molecular biology techniques

BrdU-labeled DNA immunoprecipitation and microarray analysis were performed as described previously (Lengronne et al. 2004) with the following modification. Library preparation and amplification were carried out using the GenomePlex Complete whole-genome amplification kit (Sigma). Seven micrograms of amplified DNA was fragmented using human apurinic/apyrimidinic endonuclease (APE1) in the presence of uracil DNA glycosylase and then labeled with Biotin-11-dXTPs using recombinant terminal deoxynucleotide transferase before hybridization to GeneChip Yeast Genome 2.0 arrays (Affymetrix).

### Western blotting

Protein extracts for Western blotting were prepared following cell fixation using trichloroacetic acid (Foiani et al. 1994) and were resolved by SDS–polyacrylamide gel electrophoresis. Following Western transfer, antibodies used for detection were anti-Cre recombinase (clone 2D8; Millipore), anti-Pk (clone SV5-Pk1; Serotec), and anti-α-tubulin (clone TAT-1; Crick Cell Services).

## Supplementary Material

Supplemental Material

## References

[MARIEZCURRENAGAD305557C1] Baranello L, Wojtowicz D, Cui K, Devaiah BN, Chung HJ, Chan-Salis KY, Guha R, Wilson K, Zhang X, Zhang H, 2016 RNA polymerase II regulates topoisomerase 1 activity to favor efficient transcription. Cell 165: 357–371. 27058666 10.1016/j.cell.2016.02.036 PMC4826470

[MARIEZCURRENAGAD305557C2] Baxter J, Diffley JF. 2008 Topoisomerase II inactivation prevents the completion of DNA replication in budding yeast. Mol Cell 30: 790–802. 18570880 10.1016/j.molcel.2008.04.019

[MARIEZCURRENAGAD305557C3] Baxter J, Sen N, Martínez VL, De Carandini ME, Schvartzman JB, Diffley JFX, Aragón L. 2011 Positive supercoiling of mitotic DNA drives decatenation by topoisomerase II in eukaryotes. Science 331: 1328–1332. 21393545 10.1126/science.1201538

[MARIEZCURRENAGAD305557C4] Bi X, Broach JR. 1997 DNA in transcriptionally silent chromatin assumes a distinct topology that is sensitive to cell cycle progression. Mol Cell Biol 17: 7077–7087. 9372939 10.1128/mcb.17.12.7077 PMC232564

[MARIEZCURRENAGAD305557C5] Brill SJ, DiNardo S, Voelkel-Meiman K, Sternglanz R. 1987 Need for DNA topoisomerase activity as a swivel for DNA replication for transcription of ribosomal DNA. Nature 326: 414–416. 2436053 10.1038/326414a0

[MARIEZCURRENAGAD305557C6] Cebrián J, Castán A, Martínez V, Kadomatsu-Hermosa MJ, Parra C, Fernández-Nestosa MJ, Schaerer C, Hernández P, Krimer DB, Schvartzman JB. 2015 Direct evidence for the formation of precatenanes during DNA replication. J Biol Chem 290: 13725–13735. 25829493 10.1074/jbc.M115.642272 PMC4447951

[MARIEZCURRENAGAD305557C7] Champoux JJ, Been MD. 1980 Topoisomerases and the swivel problem. In Mechanistic studies of DNA replication and genetic recombination: ICN-UCLA symposia on molecular and cellular biology (ed. Albert B), pp. 809–815. Academic, New York.

[MARIEZCURRENAGAD305557C8] Chang C-R, Wu C-S, Hom Y, Gartenberg MR. 2005 Targeting of cohesin by transcriptionally silent chromatin. Genes Dev 19: 3031–3042. 16319193 10.1101/gad.1356305 PMC1315406

[MARIEZCURRENAGAD305557C9] Charbin A, Bouchoux C, Uhlmann F. 2014 Condensin aids sister chromatid decatenation by topoisomerase II. Nucl Acids Res 42: 340–348. 24062159 10.1093/nar/gkt882 PMC3874195

[MARIEZCURRENAGAD305557C10] Cuvier O, Hirano T. 2003 A role of topoisomerase II in linking DNA replication to chromosome condensation. J Cell Biol 160: 645–655. 12604590 10.1083/jcb.200209023 PMC2173373

[MARIEZCURRENAGAD305557C11] D'Ambrosio C, Kelly G, Shirahige K, Uhlmann F. 2008a Condensin-dependent rDNA decatenation introduces a temporal pattern to chromosome segregation. Curr Biol 18: 1084–1089. 18635352 10.1016/j.cub.2008.06.058

[MARIEZCURRENAGAD305557C12] D'Ambrosio C, Schmidt CK, Katou Y, Kelly G, Itoh T, Shirahige K, Uhlmann F. 2008b Identification of *cis*-acting sites for condensin loading onto budding yeast chromosomes. Genes Dev 22: 2215–2227. 18708580 10.1101/gad.1675708 PMC2518811

[MARIEZCURRENAGAD305557C13] Dewar JM, Budzowska M, Walter JC. 2015 The mechanism of DNA replication termination in vertebrates. Nature 525: 345–350. 26322582 10.1038/nature14887 PMC4575634

[MARIEZCURRENAGAD305557C14] Díaz-Ingelmo O, Martínez-García B, Segura J, Valdés A, Roca J. 2015 DNA topology and global architecture of point centromeres. Cell Rep 13: 667–677. 26489472 10.1016/j.celrep.2015.09.039

[MARIEZCURRENAGAD305557C15] DiNardo S, Voelkel K, Sternglanz R. 1984 DNA topoisomerase II mutant of *Saccharomyces cerevisiae*: topoisomerase II is required for segregation of daughter molecules at the termination of DNA replication. Proc Natl Acad Sci 81: 2616–2620. 6326134 10.1073/pnas.81.9.2616 PMC345120

[MARIEZCURRENAGAD305557C16] Downes CS, Mullinger AM, Johnson RT. 1991 Inhibitors of DNA topoisomerase II prevent chromatid separation in mammalian cells but do not prevent exit from mitosis. Proc Natl Acad Sci 88: 8895–8899. 1656458 10.1073/pnas.88.20.8895 PMC52617

[MARIEZCURRENAGAD305557C17] Farcas A-M, Uluocak P, Helmhart W, Nasmyth K. 2011 Cohesin's concatenation of sister DNAs maintains their intertwining. Mol Cell 44: 97–107. 21981921 10.1016/j.molcel.2011.07.034 PMC3240746

[MARIEZCURRENAGAD305557C18] Foiani M, Marini F, Gamba D, Lucchini G, Plevani P. 1994 The B subunit of the DNA polymerase a-primase complex in *Saccharomyces cerevisiae* executes an essential function at the initial stage of DNA replication. Mol Cell Biol 14: 923–933. 8289832 10.1128/mcb.14.2.923-933.1994 PMC358447

[MARIEZCURRENAGAD305557C19] Gartenberg MR, Wang JC. 1992 Positive supercoiling of DNA greatly diminishes mRNA synthesis in yeast. Proc Natl Acad Sci 89: 11461–11465. 1333610 10.1073/pnas.89.23.11461 PMC50571

[MARIEZCURRENAGAD305557C20] Gerlich D, Beaudouin J, Kalbfuss B, Daigle N, Eils R, Ellenberg J. 2003 Global chromosome positions are transmitted through mitosis in mammalian cells. Cell 112: 751–764. 12654243 10.1016/s0092-8674(03)00189-2

[MARIEZCURRENAGAD305557C21] Goshima G, Yanagida M. 2000 Establishing biorientation occurs with precocious separation of the sister kinetochores, but not the arms, in the early spindle of budding yeast. Cell 100: 619–633. 10761928 10.1016/s0092-8674(00)80699-6

[MARIEZCURRENAGAD305557C22] Hawkins M, Retkute R, Müller CA, Saner N, Tanaka TU, de Moura AP, Nieduszynski CA. 2013 High-resolution replication profiles define the stochastic nature of genome replication initiation and termination. Cell Rep 5: 1132–1141. 24210825 10.1016/j.celrep.2013.10.014 PMC3898788

[MARIEZCURRENAGAD305557C23] Heun P, Laroche T, Shimada K, Furrer P, Gasser SM. 2001 Chromosome dynamics in the yeast interphase nucleus. Science 294: 2181–2186. 11739961 10.1126/science.1065366

[MARIEZCURRENAGAD305557C24] Holm C, Goto T, Wang JC, Botstein D. 1985 DNA topoisomerase II is required at the time of mitosis in yeast. Cell 41: 553–563. 2985283 10.1016/s0092-8674(85)80028-3

[MARIEZCURRENAGAD305557C25] Jeppsson K, Carlborg KK, Nakato R, Berta DG, Lilienthal I, Kanno T, Lindqvist A, Brink MC, Dantuma NP, Katou Y, 2014 The chromosomal association of the Smc5/6 complex depends on cohesion and predicts the level of sister chromatid entanglement. PLoS Genet 10: e1004680. 25329383 10.1371/journal.pgen.1004680 PMC4199498

[MARIEZCURRENAGAD305557C26] Joshi RS, Pina B, Roca J. 2010 Positional dependence of transcriptional inhibition by DNA torsional stress in yeast chromosomes. EMBO J 29: 740–748. 20057354 10.1038/emboj.2009.391 PMC2805846

[MARIEZCURRENAGAD305557C27] Kobayashi T. 2003 The replication fork barrier site forms a unique structure with Fob1p and inhibits the replication fork. Mol Cell Biol 23: 9178–9188. 14645529 10.1128/MCB.23.24.9178-9188.2003 PMC309713

[MARIEZCURRENAGAD305557C28] Koshland D, Hartwell LH. 1987 The structure of sister minichromosome DNA before anaphase in *Saccharomyces cerevisiae*. Science 238: 1713–1716. 3317838 10.1126/science.3317838

[MARIEZCURRENAGAD305557C29] Lengronne A, Katou Y, Mori S, Yokobayashi S, Kelly GP, Itoh T, Watanabe Y, Shirahige K, Uhlmann F. 2004 Cohesin relocation from sites of chromosomal loading to places of convergent transcription. Nature 430: 573–578. 15229615 10.1038/nature02742 PMC2610358

[MARIEZCURRENAGAD305557C30] Lucas I, Germe T, Chevrier-Miller M, Hyrien O. 2001 Topoisomerase II can unlink replicating DNA by precatenane removal. EMBO J 20: 6509–6519. 11707421 10.1093/emboj/20.22.6509 PMC125741

[MARIEZCURRENAGAD305557C31] McGuffee SR, Smith DJ, Whitehouse I. 2013 Quantitative, genome-wide analysis of eukaryotic replication inititaion and termination. Mol Cell 50: 123–135. 23562327 10.1016/j.molcel.2013.03.004 PMC3628276

[MARIEZCURRENAGAD305557C32] Nishimura K, Fukagawa T, Takisawa H, Kakimoto T, Kanemaki M. 2009 An auxin-based degron system for the rapid depletion of proteins in nonplant cells. Nat Methods 6: 917–922. 19915560 10.1038/nmeth.1401

[MARIEZCURRENAGAD305557C33] Ocampo-Hafalla MT, Katou Y, Shirahige K, Uhlmann F. 2007 Displacement and re-accumulation of centromeric cohesin during transient pre-anaphase centromere splitting. Chromosoma 116: 531–544. 17763979 10.1007/s00412-007-0118-4 PMC2075529

[MARIEZCURRENAGAD305557C34] O'Reilly N, Charbin A, Lopez-Serra L, Uhlmann F. 2012 Facile synthesis of budding yeast a-factor and its use to synchronize cells of a mating type. Yeast 29: 233–240. 22641466 10.1002/yea.2906

[MARIEZCURRENAGAD305557C35] Peter BJ, Ullsperger C, Hiasa H, Marians KJ, Cozzarelli NR. 1998 The structure of supercoiled intermediates in DNA replication. Cell 94: 819–827. 9753328 10.1016/s0092-8674(00)81740-7

[MARIEZCURRENAGAD305557C36] Reid RJD, Sunjevaric I, Kedacche M, Rothstein R. 2002 Efficient PCR-based gene disruption in *Saccharomyces* strains using intergenic primers. Yeast 19: 319–328. 11870855 10.1002/yea.817

[MARIEZCURRENAGAD305557C37] Rose MD, Winston F, Hieter P. 1990 Laboratory course manual for methods in yeast genetics. Cold Spring Harbor Laboratory Press, Cold Spring Harbor, NY.

[MARIEZCURRENAGAD305557C38] Schultz MC, Brill SJ, Ju Q, Sternglanz R, Reeder RH. 1992 Topoisomerases and yeast rRNA transcription: negative supercoiling stimulates initiation and topoisomerase activity is required for elongation. Genes Dev 6: 1332–1341. 1321070 10.1101/gad.6.7.1332

[MARIEZCURRENAGAD305557C39] Sogo JM, Stasiak A, Martínez-Robles ML, Krimer DB, Hernández P, Schvartzman JB. 1999 Formation of knots in partially replicated DNA molecules. J Mol Biol 286: 637–643. 10024438 10.1006/jmbi.1998.2510

[MARIEZCURRENAGAD305557C40] Spell RM, Holm C. 1994 Nature and distribution of chromosomal intertwinings in *Saccharomyces cerevisiae*. Mol Cell Biol 14: 1465–1476. 8289822 10.1128/mcb.14.2.1465-1476.1994 PMC358502

[MARIEZCURRENAGAD305557C41] Sundin O, Varshavsky A. 1980 Terminal stages of SV40 DNA replication proceed via multiply intertwined catenated dimers. Cell 21: 103–114. 6250706 10.1016/0092-8674(80)90118-x

[MARIEZCURRENAGAD305557C42] Thorpe HM, Wilson SE, Smith MCM. 2000 Control of the directionality in the site-specific recombination system of the *Streptomyces* phage phiC31. Mol Microbiol 38: 232–241. 11069650 10.1046/j.1365-2958.2000.02142.x

[MARIEZCURRENAGAD305557C43] Uemura T, Ohkura H, Adachi Y, Morino K, Shiozaki K, Yanagida M. 1987 DNA topoisomerase II is required for condensation and separation of mitotic chromosomes in *S. pombe*. Cell 50: 917–925. 3040264 10.1016/0092-8674(87)90518-6

[MARIEZCURRENAGAD305557C44] van Loenhout MTJ, de Grunt MV, Dekker C. 2012 Dynamics of DNA supercoils. Science 338: 94–97. 22983709 10.1126/science.1225810

[MARIEZCURRENAGAD305557C45] van Werven FJ, Neuert G, Hendrick N, Lardenois A, Buratowski S, van Oudenaarden A, Primig M, Amon A. 2012 Transcription of two long noncoding RNAs mediates mating-type control of gametogenesis in budding yeast. Cell 150: 1170–1181. 22959267 10.1016/j.cell.2012.06.049 PMC3472370

[MARIEZCURRENAGAD305557C46] Vig BK. 1981 Sequence of centromere separation: analysis of mitotic chromosomes in man. Hum Genet 57: 247–252. 7250964 10.1007/BF00278937

[MARIEZCURRENAGAD305557C47] Vologodskii AV, Zhang W, Rybenkov VV, Podtelezhnikov AA, Subramanian D, Griffith JD, Cozzarelli NR. 2001 Mechanism of topology simplification by type II DNA topoisomerases. Proc Natl Acad Sci 98: 3045–3049. 11248029 10.1073/pnas.061029098 PMC30604

[MARIEZCURRENAGAD305557C48] Wang LH-C, Schwarzbraun T, Speicher MR, Nigg EA. 2008a Persistence of DNA threads in human anaphase cells suggests late completion of sister chromatid decatenation. Chromosoma 117: 123–135. 17989990 10.1007/s00412-007-0131-7 PMC2755729

[MARIEZCURRENAGAD305557C49] Wang X, Reyes-Lamothe R, Sherratt DJ. 2008b Modulation of *Escherichia coli* sister chromosome cohesion by topoisomerase IV. Genes Dev 22: 2426–2433. 18765793 10.1101/gad.487508 PMC2532930

[MARIEZCURRENAGAD305557C50] Woodward J, Taylor GC, Soares DC, Boyle S, Sie D, Read D, Chathoth K, Vukovic M, Tarrats N, Jamieson D, 2016 Condensin II mutation causes T-cell lymphoma through tissue-specific genome instability. Genes Dev 30: 2173–2186. 27737961 10.1101/gad.284562.116 PMC5088566

