# Supplementary material for: Observation of DNA intertwining along authentic budding yeast chromosomes
Source: Genes Dev. 2017 Nov 1;31(21):2151–61. doi: 10.1101/gad.305557.117 (PMC5749163; doi:10.1101/gad.305557.117)
Supplement: Supplemental Material [file supp_31_21_2151__index.html]

Observation of DNA intertwining along authentic budding yeast chromosomes — Supplemental Material 

# Observation of DNA intertwining along authentic budding yeast chromosomes

## Supplemental Material

- Supplemental\_Material.pdf
