# Supplementary material for: Observation of DNA intertwining along authentic budding yeast chromosomes
Source: Genes Dev. 2017 Nov 1;31(21):2151–61. doi: 10.1101/gad.305557.117 (PMC5749163; doi:10.1101/gad.305557.117)
Supplement: Supplemental Material [file supp_gad.305557.117_Supplemental_Material.pdf]

Supplemental Material

Observation of DNA intertwining along authentic budding yeast chromosomes

Ainhoa Mariezcurrena and Frank Uhlmann

	Page
**Supplemental Figures S1 – S5**	**2**
**Supplemental Figure Legends**	**7**
**Supplemental Tables S1 – S2**	**9**

Mariezcurrena_Supplemental_Fig.S1

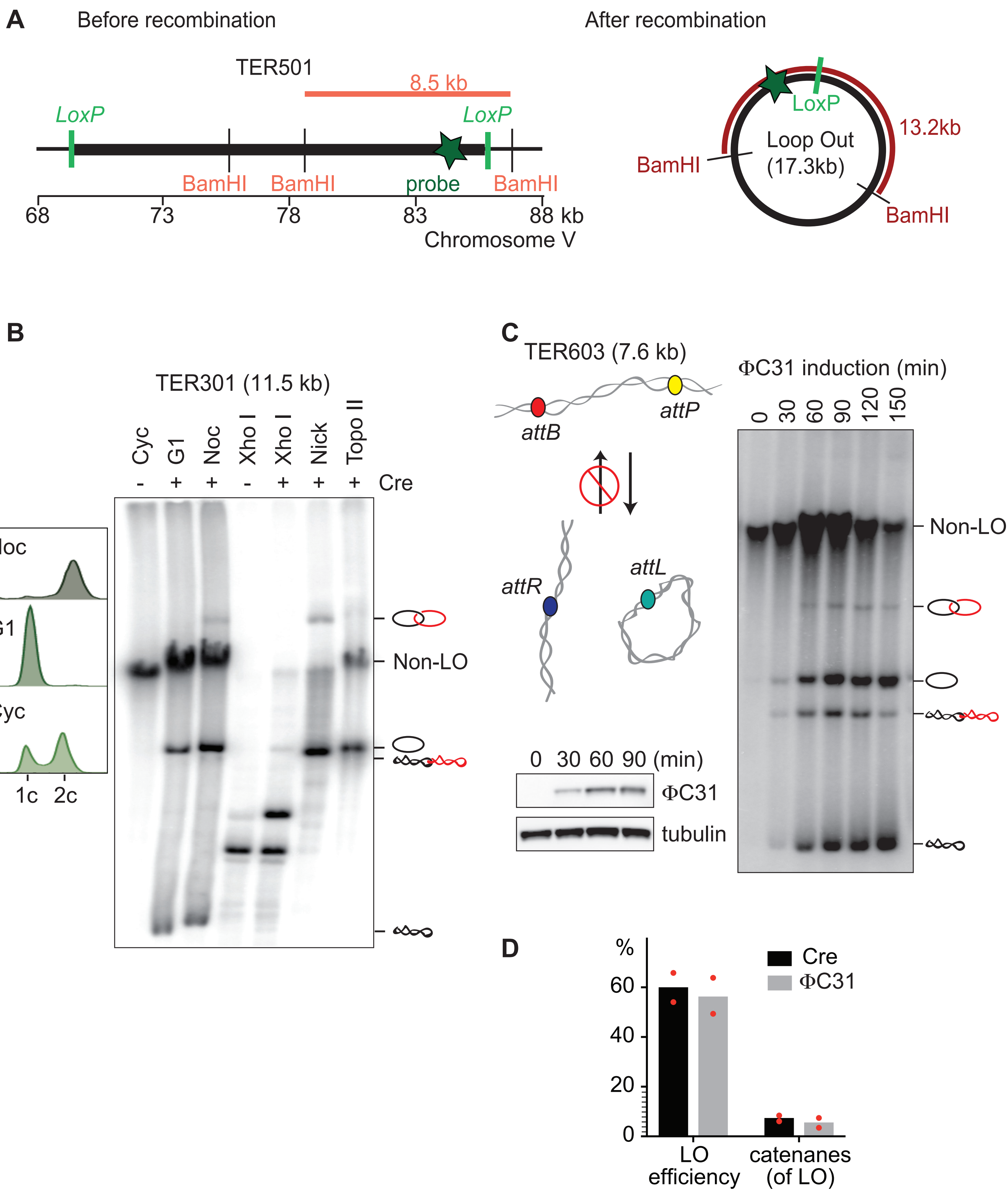

Supplemental Figure S2

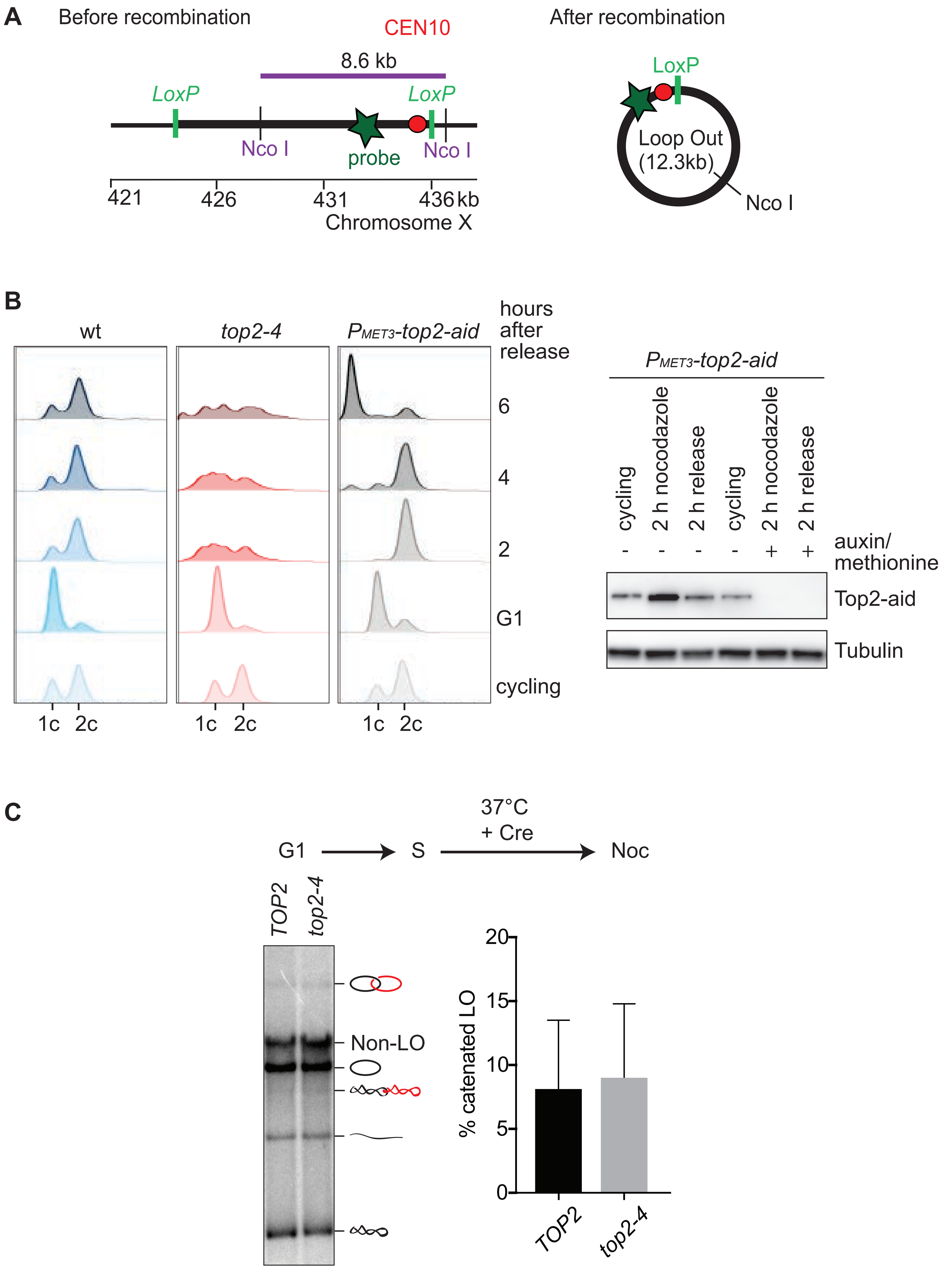

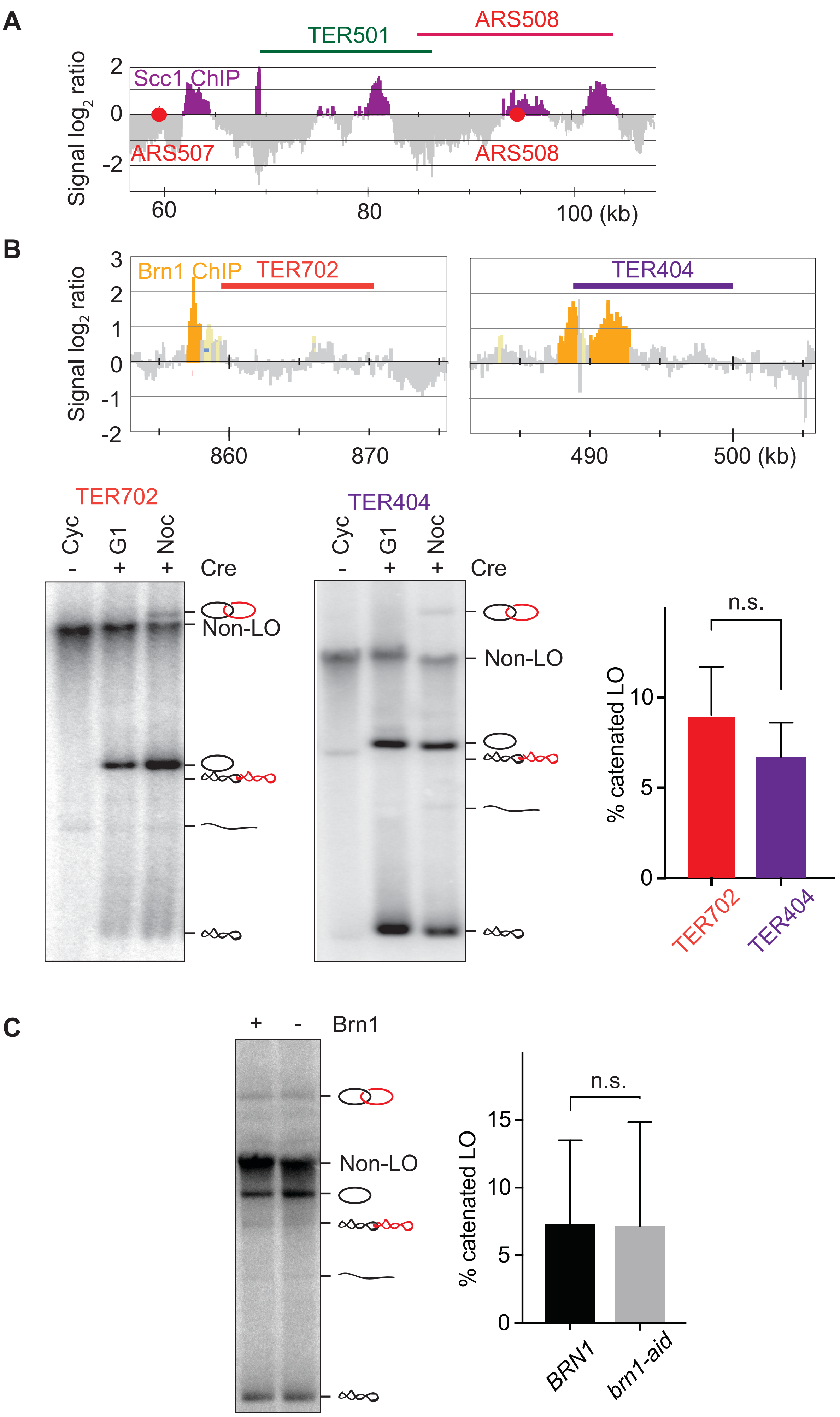
A
TER501
ARS508
Scc1 ChIP
Signal log2 ratio
2
0
-2
ARS507
ARS508
60
80
100 (kb)
B
Brn1 ChIP
TER702
TER404
Signal log2 ratio
3
2
1
0
-1
-2
860
870
490
500 (kb)
TER702
Cyc
G1
Noc
-
+
+
Cre
Non-LO
TER404
Cyc
G1
Noc
-
+
+
Cre
Non-LO
n.s.
% catenated LO
10
5
0
TER702
TER404
C
+
-
Brn1
Non-LO
n.s.
% catenated LO
15
10
5
0
BRN1
brn1-aid

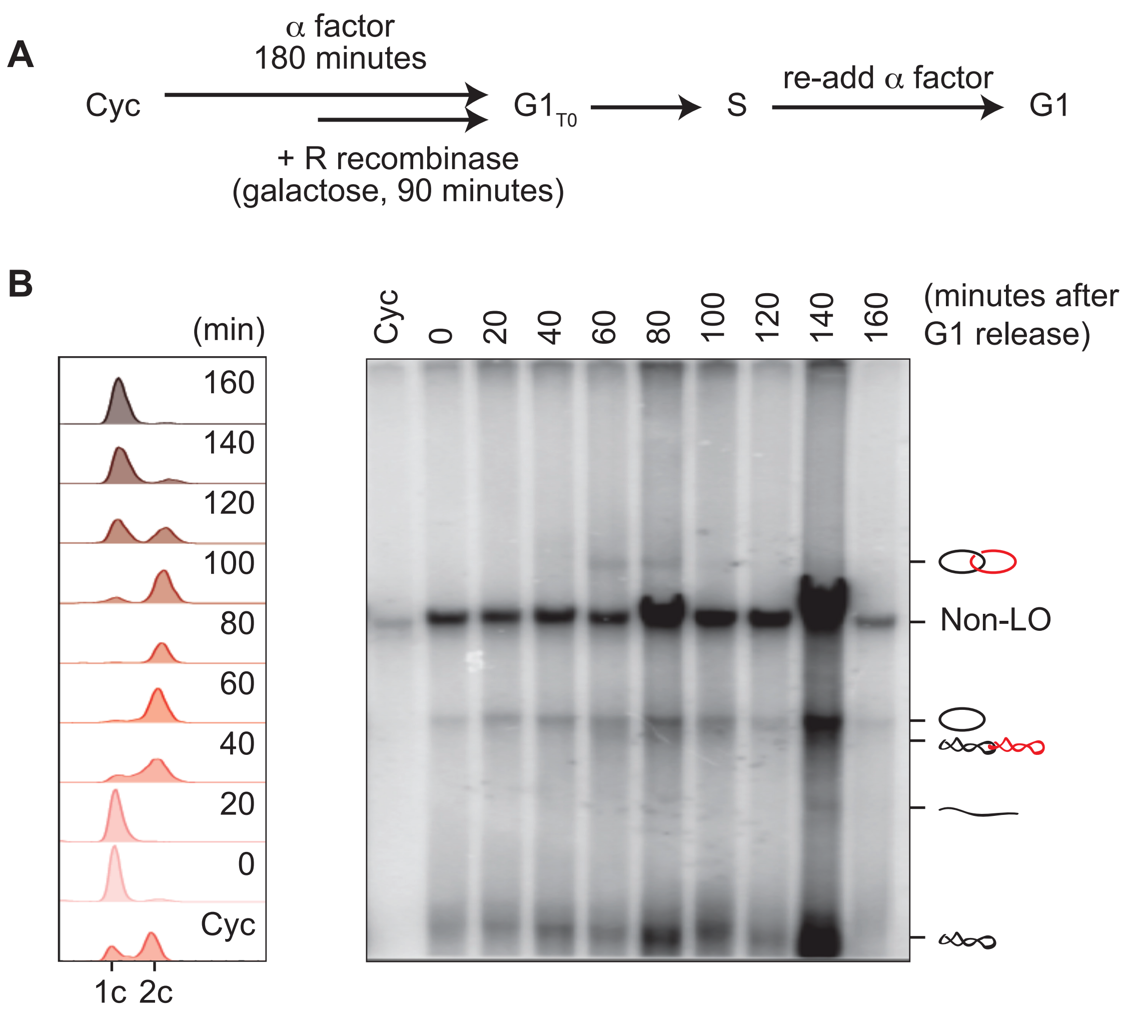
A
α factor
180 minutes
Cyc
G1T0
S
re-add α factor
G1
+ R recombinase
(galactose, 90 minutes)
B
(min)
160
140
120
100
80
60
40
20
0
Cyc
1c 2c
Cyc
0
20
40
60
80
100
120
140
160
(minutes after
G1 release)
Non-LO

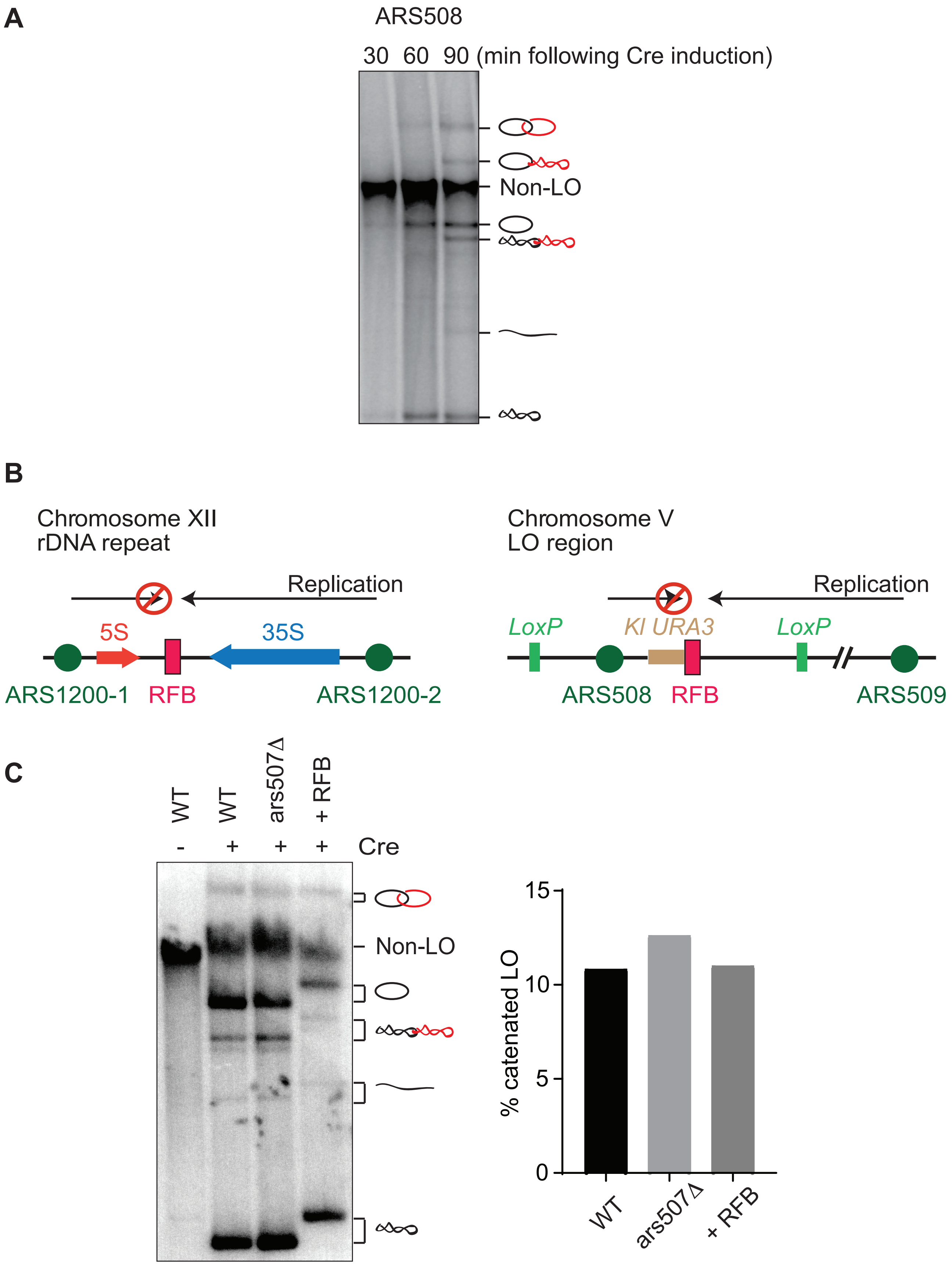
A
ARS508
30 60 90 (min following Cre induction)
Non-LO
B
Chromosome XII
rDNA repeat
Replication
5S
35S
ARS1200-1
RFB
ARS1200-2
Chromosome V
LO region
Replication
LoxP
KI URA3
LoxP
ARS508
RFB
ARS509
C
WT
WT
ars507Δ
+ RFB
- + + + Cre
Non-LO
15
10
5
0
% catenated LO
WT
ars507Δ
+ RFB

Supplemental Figure Legends

Figure S1. Topological analysis of chromosomal loop outs generated by site-specific recombination. (*A*) Schematic of the TER501 region before and after excision, including diagnostic BamHI restriction sites used for Southern analysis. (*B*) Topological analysis of an excised region surrounding TER301 on chromosome III, both from G1 and G2/M (Noc) arrested cells, together with enzyme treatments to assign band identities. FACS analysis of DNA content confirmed cell cycle synchrony. (*C*) Schematic of unidirectional ΦC31-mediated recombination between *attB* and *attP* recombinase recognition sites, surrounding TER603, and a time course of ΦC31 recombinase induction in cells arrested in G2/M by nocodazole treatment. A western blot against the Pk epitope fused to the ΦC31 recombinase is shown, tubulin served as a loading control. Loop out topology was analyzed at 30 minute intervals following ΦC31 induction. (D) Quantification of loop out efficiency and the fraction of catenated loop outs after ΦC31 recombinase induction. This is compared to TER603 loop out, flanked by *loxP* recognition sites, following Cre recombinase expression (compare also Figure 4). The means and datapoints from two independent experiments are shown.

Figure S2. Looped out catenanes reflect chromosome topology prior to excision. (*A*) Schematic of the CEN10 region analyzed in Figures 2B and C, before and after excision, including the diagnostic NcoI restriction sites. (B) Confirmation of topo II inactivation using an auxin-inducible degron and the *top2-4* allele. Cells were synchronized in G1 by α-factor treatment and samples for FACS analysis of DNA content were taken at the indicated times following release. Chromosome missegregation at the first cell division in *top2-4* cells confirms rapid topo II inactivation. In contrast, following topo II depletion by promoter shut-off and auxin addition, ploidy is maintained for the duration of approximately two cell cycles (4 hours), before missegregation ensues (6 hours). This is despite the fact that topo II becomes undetectable by western blotting using an antibody against the degron tag, two hours following release from G1 into medium containing, or not, nocodazole. Tubulin served as a loading control. (C) Schematic of the experiment in which topo II was inactivated using the temperature sensitive *top2-4* allele following completion of DNA replication but before induction of Cre recombinase expression to excise a region surrounding ARS508. A representative blot that analyzes the loop out topology is shown together with the quantification of catenated loop outs from three independent experiments. The means +/- standard error are shown.

Figure S3. The roles of cohesin and condensin. (*A*) Cohesin distribution is shown along a section of chromosome V encompassing the TER501 and ARS508 regions. The cohesin ChIP data is from (Ocampo-Hafalla et al. 2007). DNA enrichment in the Scc1 chromatin immunoprecipitates relative to a whole genome DNA sample is shown. Genomic features are indicated. (*B*) Condensin distributions surrounding the condensin-rich TER702 and condensin-poor TER404 regions are shown. The condensin ChIP data is from (D'Ambrosio et al. 2008). Bins of 16 adjacent oligonucleotide probes with a detection p value < 0.0001 are highlighted in yellow. Examples of blots that analyze the loop out topology at both regions, following excision in cells arrest in G1 using α-factor or G2/M by nocodazole (Noc) treatment, are shown. The fraction of catenated loop outs was quantified in five independent repeats of the experiment, the means +/- standard error are shown. An unpaired t-test revealed no significant difference in the fraction of catenated loop outs in both regions. (*C*) Cells were grown in medium lacking methionine and shifted to methionine-containing medium to repress expression of the condensin subunit Brn1 under control of the *MET3* promoter. 1 μM of the auxin indole-3-acetic acid (IAA) was also added, 2 hours before release from α-factor block, to induce Brn1 degradation due to its fusion to an auxin-inducible degron tag. Successful Brn1 depletion was confirmed by the characteristic FACS profile of DNA content in an aliquot of cells that were allowed to progress through mitosis (not shown; Charbin et al. 2014). For excision of the ARS508 region, cells were arrested in G2/M by nocodazole treatment before Cre recombinase induction. An example blot to analyze loop out topology is shown, together with quantification of the fraction of catenated loop outs in four independent experiments. The means +/- standard error are shown. An unpaired t-test revealed no significant difference in the fraction of catenated loop outs in the presence or absence of condensin.

Figure S4. Catenanes become detectable on the excised *HMR* locus during S phase. (*A*) Schematic of the experiment to excise the HMR locus during G1, followed by synchronous S phase before re-arrest in the following G1. (*B*) FACS analysis of DNA content to monitor cell cycle progression and the blot to analyze loop out topology during the sequential stages of the cell cycle.

Figure S5. Intertwine formation and retention during S phase. (*A*) Analysis of DNA samples taken at 30, 60 and 90 minutes following Cre induction in cells progressing through S phase in the presence of HU shows that excision of loop outs occurs mainly between 60 and 90 minutes. (*B*) Schematic of the replication fork barrier (RFB) at the rDNA locus and its ectopic integration next to ARS508. Note that a *URA3* marker for

selection is inserted next to the RFB, thereby increasing the size of the excised region. (*C*) Topological analysis of the ARS508 loop out in cells arrested in G2/M by nocodazole treatment. Cells with and without ars507Δ, used in Figure 6, are compared side by side, as well as cell containing the RFB insertion. Quantification of the fraction of catenated loop outs revealed comparable levels of intertwinings in this region in the three strains.

Supplemental Table S1. Yeast strains used in this study.

Strain No.	Genotype
AM1	*MAT**a** ade2-1 trp1-1 can1-100 ura3-1 psi+ loxP::TER501::loxP YCplac111::P_{GAL1}-Cre::LEU2*
AM2	*MAT**a** ade2-1 trp1-1 can1-100 his3-11,15 ura3-1 psi+ loxP::ARS508::loxP YCplac111::P_{GAL1}-Cre::LEU2*
AM3	*MAT**a** can1-100 his3-11,15 ura3-1 psi+ loxP::ARS508::loxP, TRP1::P_{MET3}-top2-3miniAID::KanMX OsTIR1::ADE2 YCplac111::P_{GAL1}-Cre::LEU2*
AM4	*MAT**a** ade2-1 can1-100 his3-11,15 ura3-1 psi+ loxP::ARS508::loxP top2-4::TRP1 YCplac111::P_{GAL1}-Cre::LEU2*
AM5	*MAT**a**/MAT**a** ade2-1/ade2-1 can1-100/can1-100 his3-11,15/his3-11,15 ura3-1/ura3-1 loxP::TER501::loxP/loxP::TER501::loxP YCplac111::P_{GAL1}-Cre::LEU2*
AM6	*MAT**a** ade2-1 trp1-1 can1-100 ura3-1 psi+ loxP::TER1004-CEN10::loxP YCplac111::P_{GAL1}-Cre::LEU2*
AM7	*MAT**a** ade2-1 trp1-1 can1-100 ura3-1 psi+ loxP::TER1004-CEN10::loxP P_{MET3}-cdc20::TRP1 YCplac111::P_{GAL1}-Cre::LEU2*
AM8	*MAT**a** ade2-1 trp1-1 can1-100 ura3-1 psi+ loxP::TER603::loxP YCplac111::P_{GAL1}-Cre::LEU2*
AM9	*MAT**a** ade2-1 can1-100 his3-11,15 ura3-1, psi+ loxP::TER501::loxP P_{MET3}-scc1::TRP1 YCplac111::P_{GAL1}-Cre::LEU2*
AM10	*MAT**a** ade2-1 can1-100 his3-11,15 ura3-1, psi+ loxP::ARS508::loxP P_{MET3}-scc1-TRP1 YCplac111::P_{GAL1}-Cre::LEU2*
AM11	*MAT**a** ade2-1 trp1-1 can1-100 ura3-1 psi+ loxP::TER301::loxP YCplac111::P_{GAL1}-Cre::LEU2*
AM12	*MAT**a** ade2-1 trp1-1 can1-100 ura3-1 psi+ loxP::TER301b::loxP YCplac111::P_{GAL1}-Cre::LEU2*
AM13	*MAT**a** ade2-1 trp1-1 can1-100 ura3-1 psi+ loxP::ARS702::loxP YCplac111::P_{GAL1}-Cre::LEU2*
AM14	*MAT**a** ade2-1 trp1-1 can1-100 ura3-1 psi+ loxP::TER404::loxP YCplac111::P_{GAL1}-Cre::LEU2*
AM15	*MAT**a** trp1-1 can1-100 his3-11,15 ura3-1, psi+ loxP::ARS508::loxP Brn1-3miniAID::KanMX OsTIR1::ADE2 YCplac111::P_{GAL1}-Cre::LEU2*
AM16	*MAT**a** ade2-1 trp1-1 can1-100 his3-11, 15 psi+ ARS508::RFB::klURA3 loxP::ARS508::loxP YCplac111::P_{GAL1}-Cre::LEU2*
AM17	*MAT**a** ade2-1 trp1-1 can1-100 his3-11,15 psi+ attB::klURA3::TER603::attP::KanMX YCplac111::P_{GAL1}-PK_3-Φ31C::LEU2*
CSW10 /AM18	*MAT**a** RS::HMRE-**a**2**a**1-HMRI-TRP1-$lacO_{256}$::RS ADE2::lacR-GFP YCplac111::P_{GAL1}-R::LEU2*
AM19	*MAT**a** RS::HMRE-**a**2**a**1-HMRI-TRP1-$lacO_{256}$::RS ADE2::lacR-GFP sir2Δ::KanMX YCplac111::P_{GAL1}-R::LEU2*
AM20	*MAT**a** ade2-1 trp1-1 can1-100 his3-11,15 psi+ loxP::ARS508::loxP ars507Δ::URA3 YCplac111::P_{GAL1}-Cre::LEU2*
AM21	*MAT**a** ade2-1 trp1-1 can1-100 1 psi+ loxP::ARS508::loxP ars507Δ::URA3 P_{ADH1}-hENT1-TK::HIS3 YCplac111::P_{GAL1}-Cre::LEU2*

Supplemental Table S2. Details of loop out regions.

Locus (*Chromosome number*)	Elements contained*	Efficiency**	Upstream recombinase site *(chromosome coordinate)*	Downstream recombinase site *(chromosome coordinate)*	Size (bp)
TER501 (V)	TER, CH, CN	-0.499	69508	86765	17,257
ARS508 (V)	ARS, CH, CN,	0.919	84667	102414	17,747
TER603 (VI)	TER, CN	-0.642	176871	184501	7,630
TER603 (VI) *attB/attP (URA3)*	TER, CN	-0.642	176871	184501	9,267
CEN10 (X)	TER, CEN, CH	-0.504	425102	436770	11,668
TER301 (III)	TER, CH	-0.404	48222	66258	18,036
TER301b (III)	TER, CH,	-0.404	54930	66258	11,328
TER702 (VII)	TER	-0.453	859733	870128	10,395
TER404 (IV)	TER, CH, CN,	-0.580	489031	500957	11,926

* Elements contained in the loop outs are abbreviated, TER: replication termination region, ARS: autonomously replicating sequence/replication origin, CEN: centromere, CH: cohesin binding site, CN: condensing binding site.
** Efficiency is an estimate of the proportion of a cell population that fires a given replication origin (Efficiency: from 0 to 1), or experiences fork convergence at a given termination region (Efficiency: from 0 to -1; McGuffee et al., 2013).
